# The miRNA199a/SIRT1/P300/Yy1/sST2 signaling axis regulates adverse cardiac remodeling following MI

**DOI:** 10.1038/s41598-021-82745-9

**Published:** 2021-02-16

**Authors:** Maria Carmen Asensio-Lopez, Yassine Sassi, Fernando Soler, Maria Josefa Fernandez del Palacio, Domingo Pascual-Figal, Antonio Lax

**Affiliations:** 1 Biomedical Research Institute Virgen de la Arrixaca (IMIB-Arrixaca), University of Murcia, Murcia, Spain; 2 Cardiovascular Research Center, Icahn School of Medicine at Mount Sinai, New York, USA; 3 Biochemical and Molecular Biology Department, University of Murcia, Murcia, Spain; 4 Veterinary Teaching Hospital, Veterinary Medicine and Surgery Department, University of Murcia, Murcia, Spain; 5 Cardiology Department, Hospital Virgen de la Arrixaca, IMIB-Arrixaca, University of Murcia, Murcia, Spain; 6 Centro Nacional de Investigaciones Cardiovasculares (CNIC), Madrid, Spain; 7 CIBERCV, Madrid, Spain

**Keywords:** Cardiovascular diseases, Heart failure, Biomarkers, Preclinical research, Translational research

## Abstract

Left ventricular remodeling following myocardial infarction (MI) is related to adverse outcome. It has been shown that an up-regulation of plasma soluble ST2 (sST2) levels are associated with lower pre-discharge left ventricular (LV) ejection fraction, adverse cardiovascular outcomes and mortality outcome after MI. The mechanisms involved in its modulation are unknown and there is not specific treatment capable of lowering plasma sST2 levels in acute-stage HF. We recently identified Yin-yang 1 (Yy1) as a transcription factor related to circulating soluble ST2 isoform (sST2) expression in infarcted myocardium. However, the underlying mechanisms involved in this process have not been thoroughly elucidated. This study aimed to evaluate the pathophysiological implication of miR-199a-5p in cardiac remodeling and the expression of the soluble ST2 isoform. Myocardial infarction (MI) was induced by permanent ligation of the left anterior coronary artery in C57BL6/J mice that randomly received antimiR199a therapy, antimiR-Ctrl or saline. A model of biomechanical stretching was also used to characterize the underlying mechanisms involved in the activation of Yy1/sST2 axis. Our results show that the significant upregulation of miR-199a-5p after myocardial infarction increases pathological cardiac hypertrophy by upregulating circulating soluble sST2 levels. AntimiR199a therapy up-regulates Sirt1 and inactivates the co-activator P300 protein, thus leading to Yy1 inhibition which decreases both expression and release of circulating sST2 by cardiomyocytes after myocardial infarction. Pharmacological inhibition of miR-199a rescues cardiac hypertrophy and heart failure in mice, offering a potential therapeutic approach for cardiac failure.

## Introduction

Heart failure (HF) is one of the leading pathological causes of mortality worldwide^1,2^. Myocardial stress, due to myocardial infarction (MI), valvular heart disease or prolonged hypertension, induces pathological cardiac remodeling, which contributes to the development of HF^3,4^. In acute-stage HF elevated plasma levels of soluble ST2 (sST2), the product of the gene *IL1rl1*, is essentially a marker of inflammation, stretch and signals the presence and severity of adverse cardiac remodeling and tissue fibrosis^5–7^. However, despite its approval and the growing use of sST2 by practicing clinicians, issues related to its pathobiology remain incompletely understood, including the molecular mechanisms that regulate its expression. Indeed, there are no treatments aimed at modulating specifically sST2 expression as potential therapeutic target in HF. Recently, we pioneered in suggesting Yin-yang 1 (Yy1) as a transcription factor that is able to up-regulate sST2 expression under stress conditions, and of HDAC4 as its necessary co-repressor since it is able to block the up-regulated expression of sST2 in the setting of biomechanical strain^8^. The activity of the transcription factor Yy1 is also regulated by the pro-hypertrophic and co-activator P300 protein^9^, however, up to now, the underlying mechanism related to modulation of P300 expression and its action on the modulation of sST2 through activation of Yy1 is not totally understood.

MicroRNAs (miRNAs) are small noncoding RNAs functioning as fundamental modulators of cardiovascular homeostasis and pathophysiology; there is a growing interest in developing their potential as both biomarkers and therapeutic targets for cardiovascular pathologies^10–14^. In this context, myocardial miR-199-5p levels are upregulated under cardiac damage (i.e. MI), which contributes to the decline of the cardiac function itself. Indeed, this up-regulation increases apoptosis^15^ and inhibits cell proliferation^16^. Pharmacological inhibition of miR-199-5p attenuates hypoxia/reoxygenation-induced cytotoxicity and decreases phenylephrine-induced cardiomyocyte hypertrophy^17,18^, indicating that the inhibition of this miRNA might serve as a novel therapeutic approach for treating HF. Studies specifically designed to assess the potential role and mechanism of miR-199a in the treatment of HF are necessary.

Herein, we evaluated whether miR-199a-5p contributes to the onset of MI-induced pathological hypertrophy. Based on our previous studies and with the aim of characterizing the molecular mechanisms in this pathway, we investigated whether miR199a-5p is able to modulate the expression of circulating soluble sST2 protein following MI.

## Results

### The miR-199a-5p is dynamically regulated following MI

**Figure 1:**
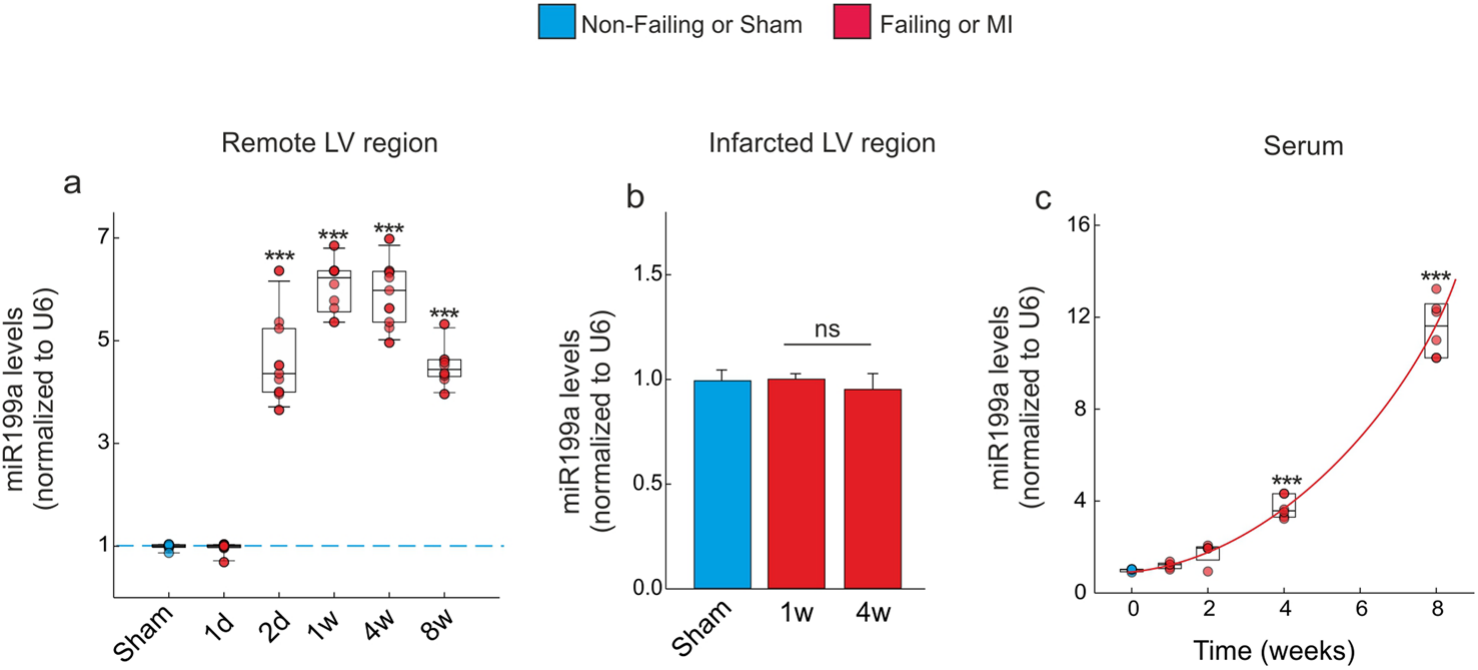
miR199a-5p is dynamically regulated following MI. (**a**) Quantification of miR199a levels by RT-qPCR in the remote LV region after MI. (**b**) Quantification of cardiac miR199a levels in the infarcted LV region by RT-qPCR. (**c**) Serum levels of miR199a after MI. n = 10 mice per groups. All quantitative data are reported as means ± SEM. ***P < 0.001 determined by two-way ANOVA followed by Bonferroni’s post hoc test. d: day(s); w: week(s).

In order to evaluate whether miR-199a-5p, hereafter described as miR-199a, is involved in MI-induced adverse cardiac remodeling, we first assessed whether miR-199a is regulated in both remote non-infarcted LV region and infarcted LV region of infarcted mice. By quantitative real time PCR analysis, we found temporal changes in miR-199a expression that occur in mice subjected to MI (Fig. 1a).

This model of cardiac damage led, 2 days after MI, to a prominent increase in cardiac miR-199a level in the remote region (p < 0.001). The increase in cardiac miR-199a level remained elevated until 4 weeks post-MI, followed by a slight down-regulation at 8 weeks (p < 0.001) (Fig. 1a). Upon isolation of CMs and CFs from the non-infarcted LV region, miR-199a were found to be significantly higher in CMs compared to CFs at 4 weeks post-MI (Supplementary Figure 2). No changes in miR-199a levels were detected in the infarcted LV region, 1- and 4-weeks post-MI (Fig. 1b). As expected, the miR-199a levels were significantly increased in mice serum at 4- and 8-weeks post MI (p < 0.001) (Fig. 1c).

### AntimiR-199a protects against MI-induced cardiac hypertrophy and fibrosis

**Figure 2:**
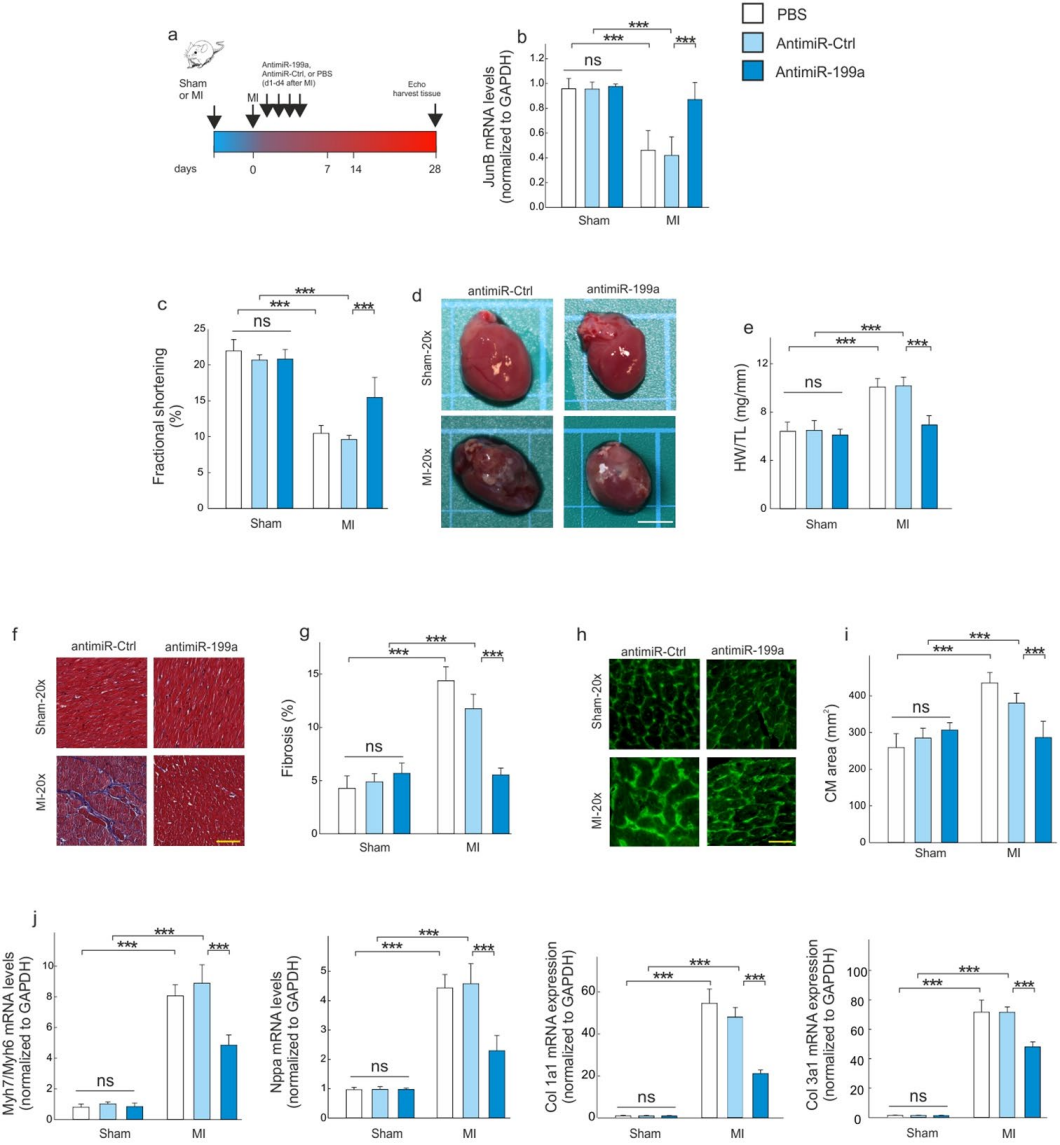
AntimiR-199a treatment protects against cardiac hypertrophy and fibrosis following MI. (**a**) Design of the study. (**b**) JunB mRNA levels in the remote non-infarcted LV region 4 weeks after MI. (**c**) Echocardiographic analysis of left ventricular fractional shortening in sham- and MI-operated mice 27 days after the first injection of antimiR-199a, antimiR-ctrl or PBS. (**d**) Representative images of hearts harvested 4 weeks post-MI and treated, or not, with antimiR-199a; Scale bar: 0.5 cm. (**e**) Heart weight-to-tibia length ratio. (**f**) Representative left ventricular sections of the indicated treatment groups stained with Masson’s trichrome; Scale bar: 25 μm. (**g**) Quantification of interstitial fibrosis. (**h**,**i**) WGA staining of left ventricular tissue from mice for determination of cardiac and CM hypertrophy; Scale bar: 25 μm. (**j**) Quantitative real-time PCR analysis of molecular markers for cardiac myocyte hypertrophy (Myh7/Myh6, Nppa) and of fibrosis-associated collagens. All quantifications derive from n = 10 mice/group, PCR performed with 2 replicates each. All quantitative data are reported as means ± SEM. ***P < 0.001 determined by two-way ANOVA followed by Bonferroni’s post hoc test. CM: cardiomyocyte; Col: collagen; Ctrl: control; HW/TL: ratio heart weight versus tibia length; MI: myocardial infarction; Myh: myosin heavy chain; Nppa: natriuretic Peptide A; WGA: wheat germ agglutinin.

Given the up-regulation of miR-199a following MI, we next sought to inhibit miR-199a acutely in order to evaluate its effects on adverse cardiac remodeling. PBS and anti-miR-Ctrl were used as controls. The efficiency of antimiR-199a was validated by measuring the expression level a miR-199a target, JunB^15^. Four consecutive injections of a modified antisense oligonucleotide, designed to target mouse miR-199a (antimiR-199a) (Fig. 2a), lead to a 53% increase in JunB mRNA levels at 4 weeks post-MI (p < 0.001) (Fig. 2b). This increase was also observed in cardiac myocytes isolated from the LV remote region. Here, data analysis showed an increase of 56% in JunB mRNA levels when antimiR-199a therapy was used (Supplementary Figure 3). Infusion of antimiR-199a protected from cardiac dysfunction, cardiac hypertrophy at the tissue and cellular level, and from fibrosis (Fig. 2c–i; and Supplementary Table 1). Likewise, the corresponding gene expression signatures revealed a decrease of markers for hypertrophy (Myh7/Myh6 and Nppa) and fibrosis (Col1a1 and Col3a1) (Fig. 2j).

### AntimiR-199a therapy prevents cardiac Sirt1 down-regulation and P300 acetylation

**Figure 3:**
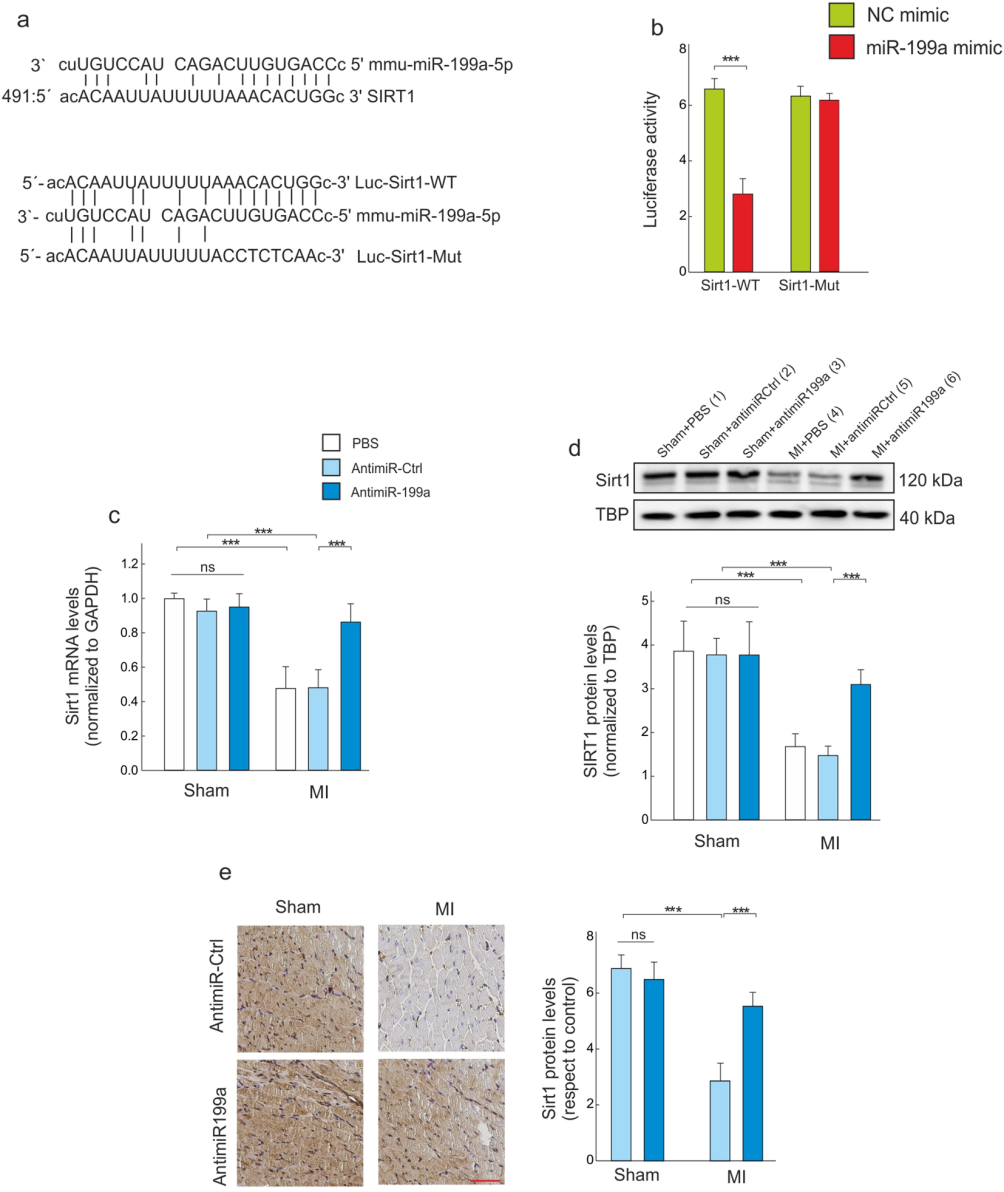
Pharmacological inhibition of miR-199a prevents Sirt1 down-regulation following MI. (**a**) Up: the putative miR-199a-5p binding sequence within the 3′-UTR of Sirt1 mRNA; Down: the sequences and position of Sirt1 containing wild type and mutant binding site of miR-199a that cloned into the luciferase reporter vector. (**b**) Dual-luciferase reporter assay. (**c**) Determination of Sirt1 mRNA levels in the non-infarcted LV region 4 weeks after MI. (**d**) Sirt1 protein levels in the non-infarcted region 4 weeks after MI; Representative full western blot images are shown in supplementary material. (**e**) Assessment of Sirt1 protein levels by immunohistochemical analysis in the non-infarcted LV; Scale bar: 25 μm. All quantifications derive from n = 10 mice/group, PCR experiments were performed with 2 replicates each. All quantitative data are reported as means ± SEM. ***P < 0.001 determined by two-way ANOVA followed by Bonferroni’s post hoc test. GAPDH: Glyceraldehyde-3-Phosphate Dehydrogenase; TBP: TATA binding protein; other abbreviations as in Fig. 2.

We next evaluated plausible targets that could be related to the cardioprotective effect of antimiR-199a therapy. Using the *in-silico* analysis tools TARGETSCAN, ENRICHR and MIRTARBASE, we confirmed Sirtuin 1 (Sirt1) as a potential target of miR-199a with a high binding score of − 1.17. The selection of Sirt 1 was also supported by its role in the regulation of P300, a coactivator involved in the transcriptional activity of the transcription factor Yy1^5^. As shown in Fig. 3a, Sirt1 3′-UTR contains putative miR-199a binding sites. To evaluate a potential binding of miR-199a on the 3′UTR of Sirt1, a luciferase reporter assay was used in HEK 293T/17 cells (Fig. 3b). For that, simultaneous transfection of miR-199a mimic or NC mimic and Sirt1 (WT or Mut) were conducted. The luciferase activity was significantly repressed upon miR-199a transfection using the wild-type 3′-UTR (p < 0.001), whereas the luciferase activity was unchanged when a mutant 3′-UTR of Sirt1 was used. These results indicate a direct binding of miR-199a to Sirt13′-UTR. We further found that miR-199a inhibition in the non-infarcted LV region significantly increased Sirt1 expression at the mRNA and protein levels (Fig. 3c–e).

**Figure 4:**
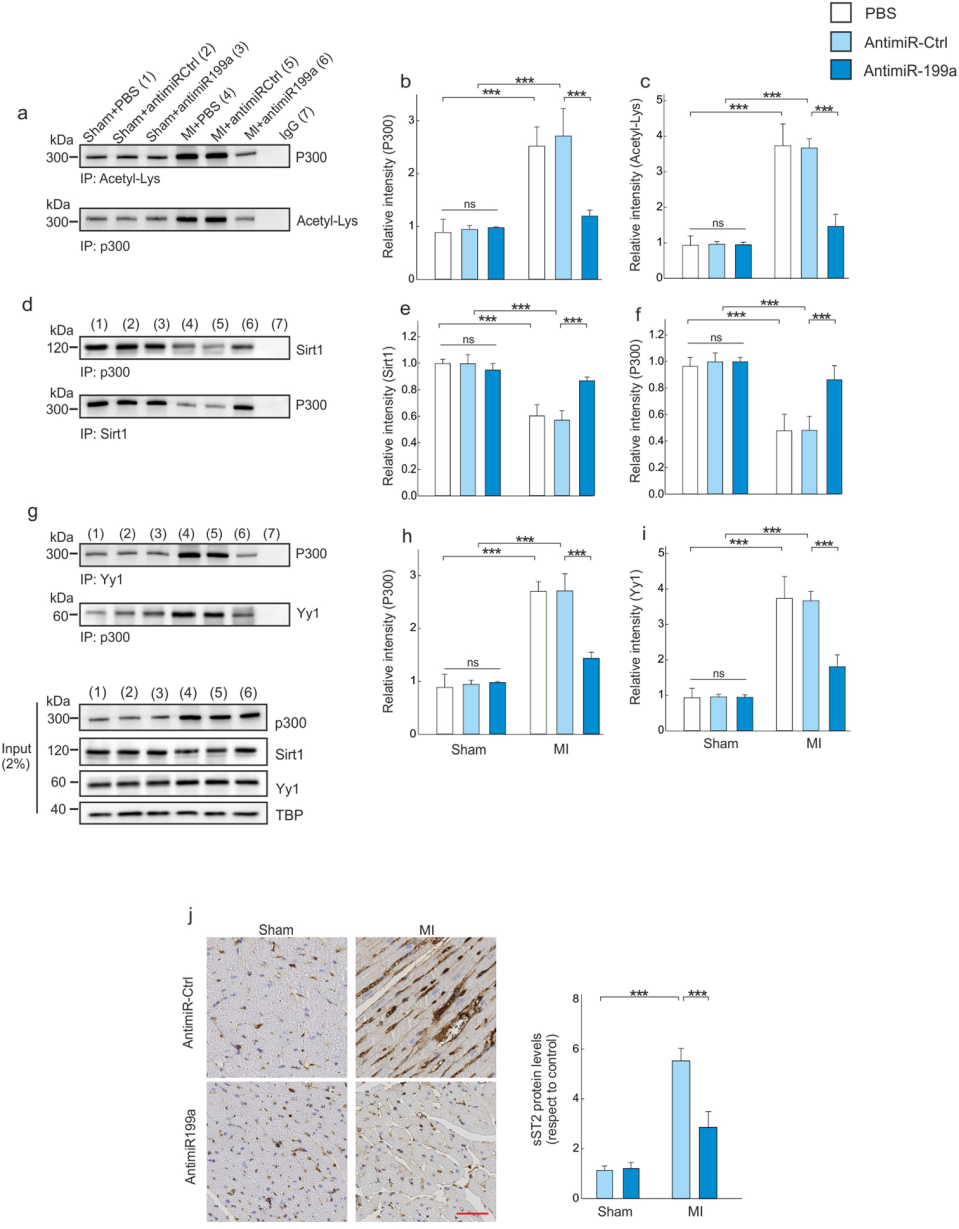
AntimiR-199a therapy prevents Sirt1 downregulation and P300 acetylation. (**a**) Co-immunoprecipitation of the P300-acetyl-Lys complex. (**b**) Densitometric analysis of the co-immunoprecipitated P300 protein. (**c**) Densitometric analysis of the co-immunoprecipitated acetyl-Lys. (**d**) Co-immunoprecipitation of the Sirt1/P300 complex. (**e**) Densitometric analysis of the co-immunoprecipitated Sirt1 protein. (**f**) Densitometric analysis of the co-immunoprecipitated P300 protein. (**g**) Co-immunoprecipitation assay of the P300/Yy1 complex. (**h**) Densitometric analysis of the co-immunoprecipitated P300 protein. (**i**) Densitometric analysis of the co-immunoprecipitated Yy1protein. (**j**) Representative immunohistochemical images and quantifications of sST2 in the remote LV region of sham and MI mice treated with antimiR-Ctrl or animiR-199a; Scale bar: 25 μm. Representative full western blot images are shown in supplementary material. All quantifications derive from n = 10 mice/group. All quantitative data are reported as means ± SEM. ***P < 0.001 determined by two-way ANOVA followed by Bonferroni’s post hoc test. Abbreviations as in Figs. 2 and 3.

In parallel, we found P300 acetylation (the active form of P300) to be increased in the non-infarcted LV region of MI mice, whereas antimiR-199a prevented this increase (Fig. 4a–c). P300 mRNA levels in the non-infarcted LV region of mice treated with antimiR-Ctrl were positively correlated with miR-199a levels (p < 0.001, r = 0.36, data not shown).

Next, through proteomic analysis of the Sirt1 interactome using the STRING database (http://string-db.org/), we determined that Sirt1 interacts with P300 in the murine heart with a score of 0.93 (Supplementary Figure 3). To confirm this interaction, LV lysates prepared from the non-infarcted LV or from the LV of sham mice were immunoprecipitated with an anti-P300 antibody and probed with an anti-Sirt1 antibody. In parallel, cited lysates were immunoprecipitated with an anti-Sirt1 antibody and probed with an anti-P300 antibody. This reciprocal immunoprecipitation experiment showed that P300 and Sirt1 are complexed in the LV of sham animals (Fig. 4d–f). MI induced a decrease in the accumulation of this complex and antimiR-199a treatment prevented this effect (Fig. 4d–f). We further examined whether the modulation of Sirt1/P300 axis by antimiR-199a synergistically regulates the transcription factor Yy1 and the expression and release of circulating soluble ST2 after MI. While in the non-infarcted LV, Yy1 protein levels were increased and not affected by antimiR-199a, we observed a direct effect on the interaction between Yy1 and P300 (Fig. 4g–i). This reciprocal immunoprecipitation experiment showed that P300 and Yy1 are complexed in the non-infarcted LV and antimiR-199a prevented this interaction (Fig. 4g–i). The effect of antimiR-199a therapy on cardiac P300-Yy1 interaction occurred with a significant decrease of sST2 expression (Fig. 4j). P300 mRNA levels in the non-infarcted LV of antimiR-Ctrl-treated mice were positively correlated with circulating soluble ST2 levels (p < 0.001, r = 0.45) (data not shown).

### An increase in antimiR199a-induced Sirt1 levels leads to a deacetylation of P300, prevents the activation Yy1 and increases circulating soluble ST2 level

**Figure 5:**
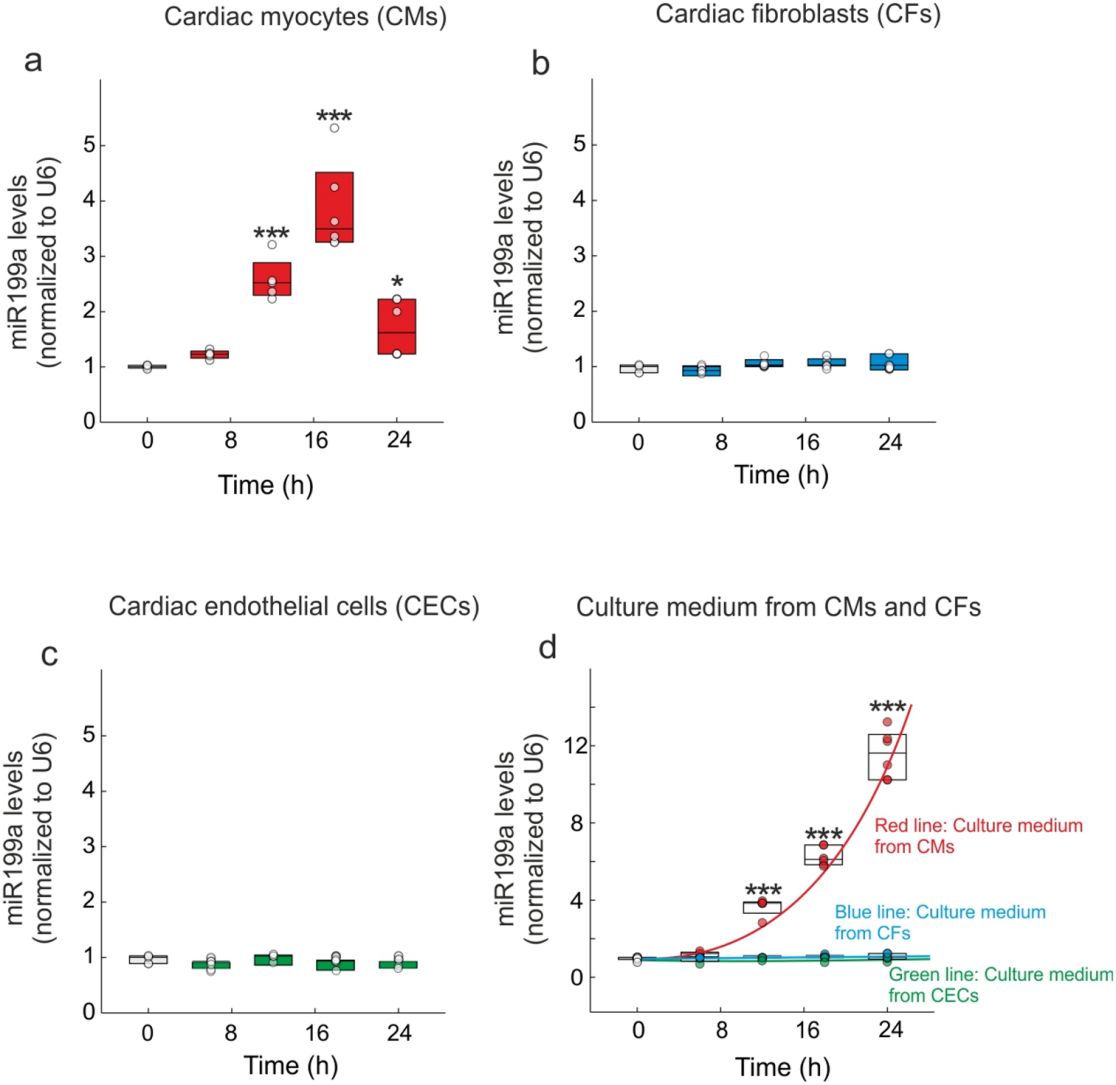
The miR199a-5p is dynamically regulated in CMs after cyclic stretching. (**a**) miR199a levels in CMs after cyclic stretching quantified by RT-qPCR. (**b**) miR199a levels in CFs after cyclic stretching quantified by RT-qPCR. (**c**) miR199a levels in ECs after cyclic stretching quantified by RT-qPCR. (**d**) miR199a levels in the supernatants of CMs (red), CFs (blue) and CECs (green) assessed by RT-qPCR after cyclic stretching. All quantifications derive from n = 10 independent assays/group. All quantitative data are reported as means ± SEM. ***P < 0.001 respect to control assay; determined by two-way ANOVA followed by Bonferroni’s post hoc test.

We next sought to identify the myocardial source of miR-199. Therefore, we subjected different cardiac cells to cyclic stretching. CMs displayed a significant increase in miR-199a levels in a time-dependent manner until 18 h post-treatment (p < 0.001), followed by a decrease 24 h after cyclic stretching (p < 0.05) (Fig. 5a).

Different results were observed in CFs and endothelial cells subjected to cyclic stretching. Indeed, stretching had no effects on the miR-199a levels in these cells (Fig. 5b,c). Furthermore, the miR-199a levels were significantly increased in the culture media of primary CMs (p < 0.01) but not in the culture medium of CFs nor endothelial cells (Fig. 5d). Therefore, we hypothesized that miR-199 levels are increased in CMs where their release is regulated upon MI.

**Figure 6:**
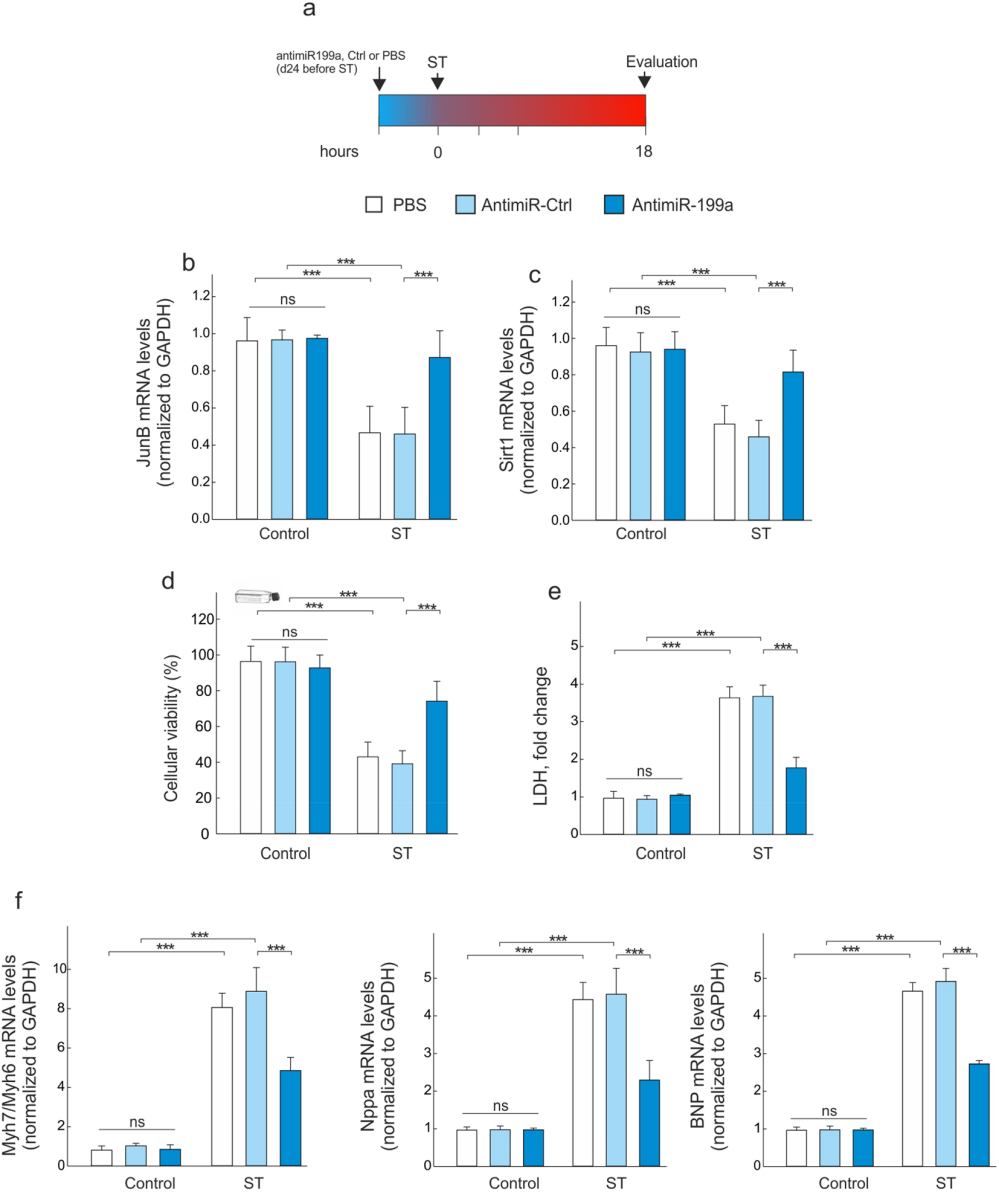
AntimiR-199a treatment prevents cardiac hypertrophy. (**a**) Design of the study using CMs under stretching. (**b**) Quantification of JunB mRNA levels in CMs after cyclic stretching assessed. (**c**) Quantification of Sirt1 mRNA levels by RT-qPCR in CMs after cyclic stretching. (**d**) Cell viability measured by MTT assay of CMs treated with PBS, antimiR-Ctrl or antimiR-199a. (**e**) LDH levels in CMs treated with PBS, antimiR-Ctrl or antimiR-199a after cyclic stretching. (**f**) Quantitative real-time PCR analysis of molecular markers for cardiac myocyte hypertrophy (Myh7/Myh6, Nppa and BNP). All quantifications derive from n = 10 independent assays/group. All quantitative data are reported as means ± SEM. ***P < 0.001 determined by two-way ANOVA followed by Bonferroni’s post hoc test. BNP: brain natriuretic peptide; LDH: lactate dehydrogenase; ST: stretching; other abbreviations as in Fig. 2.

We next sought to determine whether the cardiac protective mechanism of miR199a is mediated through the P300/Yy1/sST2 axis. As shown in Fig. 6, antimiR-199a treatment not only induced a significant up-regulation of JunB and Sirt1 mRNA levels following stretching (Fig. 6b,c), but also prevented the cardiac damage induced by stretching, as determined by cell viability (Fig. 6d), and cellular necrosis (Fig. 6e). Likewise, the corresponding gene expression signatures revealed a decrease of markers for hypertrophy (Myh7/Myh6, Nppa and BNP) (Fig. 6f).

**Figure 7:**
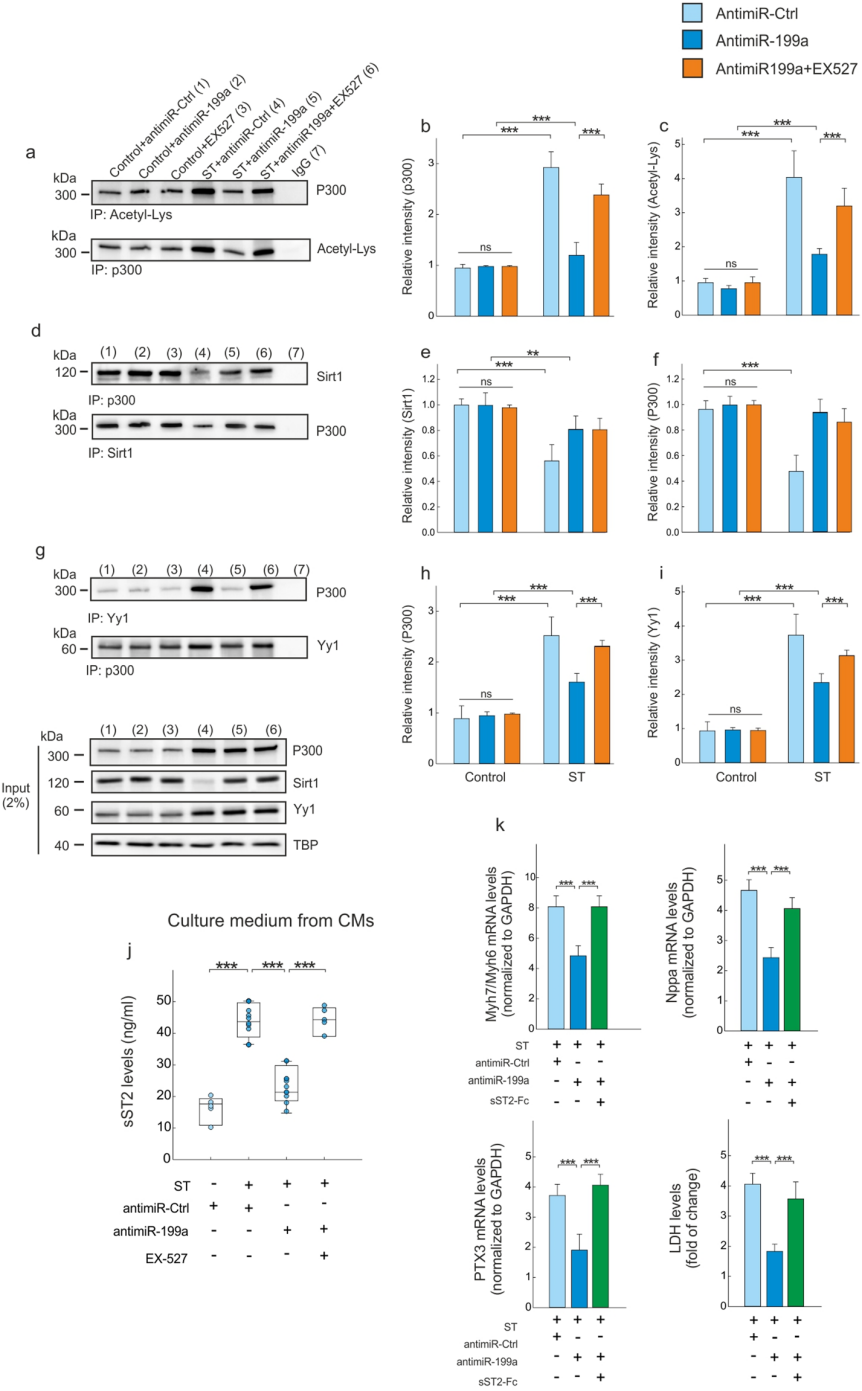
P300 deacetylation prevents Yy1 activation leading to sST2 down-regulation. (**a**) Co-immunoprecipitation of the P300-acetyl-Lys complex in control and stretched CMs that were treated with PBS, antimiR-Ctrl or antimiR-199a. (**b**) Densitometric analysis of the co-immunoprecipitated P300 protein. (**c**) Densitometric analysis of the co-immunoprecipitated acetyl-Lys. (**d**) Co-immunoprecipitation of the Sirt1/P300 complex in control and stretched CMs that were treated with PBS, antimiR-Ctrl or antimiR-199a. (**e**) Densitometric analysis of the co-immunoprecipitated Sirt1 protein. (**f**) Densitometric analysis of the co-immunoprecipitated P300 protein. (**g**) Co-immunoprecipitation assay of the P300/Yy1 complex in control and stretched CMs that were treated with PBS, antimiR-Ctrl or antimiR-199a. (**h**) Densitometric analysis of the co-immunoprecipitated P300 protein. (**i**) Densitometric analysis of the co-immunoprecipitated Yy1protein. (**j**) sST2 protein levels in supernatant from CMs under stretching. (**k**) Quantitative real-time PCR analysis of molecular markers for cardiac myocyte hypertrophy (Myh7/Myh6, Nppa) and cardiac death (PTX3, LDH); sST2-Fc (100 nM) is added 30 min after antimiR-199a therapy. Representative full western blot images are shown in supplementary material. All quantifications derive from n = 10 independent assays/group. All quantitative data are reported as means ± SEM. ***P < 0.001 determined by two-way ANOVA followed by Bonferroni’s post hoc test. PTX3: pentraxin 3; other abbreviations as in Figs. 2 and 6.

By co-immunoprecipitation, we next found EX-527 (a Sirt1 selective inhibitor) treatment to reverse the effect of miR-199 on P300 acetylation (Fig. 7a–c). Moreover, while EX-527 did not have any impact on the effect of miR-199 on the interaction between Sirt1 and P300 under stretching (Fig. 7d–f), it increased effect of miR-199 on the interaction between P300 and Yy1 (Fig. 7g–i). Finally, we sought to evaluate the effect of this modulation on the cardiac release of circulating soluble ST2. The expression and secretion of sST2 were increased in CMs under stretching, and these effects were decreased by antimiR-199a (Fig. 7j). EX-527 treatment blocked the effect of antimiR199a on the sST2 secretion (p < 0.001) (Fig. 7j). To obtain more direct evidence that the beneficial effect of antimiR199a therapy is related to down-regulation of the cardiac sST2 expression, we next examined whether incubation with the soluble extracellular domain of ST2 coupled to the Fc fragment of human IgG1 (sST2/Fc) could interfere with the cardioprotective effect of antimiR-199a therapy. As showed in Fig. 7k, sST2/Fc treatment is able to prevent the cardioprotective effect of antimiR-199a.

## Discussion

Cardiac adverse hypertrophy remains the major risk factor for heart failure, which represents one of the leading causes of re-hospitalization and death in general population^17,19^. In this context, cardiomyocyte necrosis and mechanical stretching cause the release of circulating soluble sST2 isoform^8,20,21^, a key biomarker used to diagnose cardiac disease conditions accurately and to effectively prognosticate the high-risk patients. Thus, higher levels of circulating soluble sST2 are associated with increased myocardial fibrosis, adverse cardiac remodeling, and worse cardiovascular outcomes^5–7,22^. However, despite its approval and the growing use of sST2 by practicing clinicians, issues related to the molecular mechanisms that regulate its expression after MI remain incompletely understood, including the identification of which factors are implicated in the transcription to generate the sST2 protein in cardiac tissue. In a study recently published by our research group, we identified Yy1 as a transcription factor that is able to up-regulate sST2 expression under biomechanical strain and HDAC4 as its necessary co-repressor since it is able to block the upregulated expression of sST2 in basal conditions^8^. Here, we identified a novel molecular pathway implicated in the regulation of the transcription factor Yy1 and on the expression and release of soluble sST2 in the infarcted myocardium. In this study, we demonstrated for the first time that pharmacological inhibition of miR-199a-5p in mouse model for MI, prevents the acetylation of the pro-hypertrophic protein P300, thus leading to protect the heart against adverse cardiac remodeling. AntimiR-199a-induced Sirt1 upregulation inactivating the co-activator P300, leading to Yy1 inhibition and finally decreasing both expression and release of circulating soluble ST2 by CMs after MI.

Several studies reported miR-199a-5p to be upregulated during adverse cardiac hypertrophy, thus affecting the cardiac function^23–25^. Indeed, while miR199a-5p overexpression induces cardiomyocyte proliferation and enlarges cell size^23,26^, its downregulation reduces phenylephrine-induced cardiomyocyte hypertrophy in vitro^26^. In a recently published study, the authors established that miR-199a-5p could represent a potential therapeutic target for the treatment of MI^27^. It has also been reported that one of the mechanisms by which atorvastatin prevents ischemia–reperfusion-induced cardiac injury is through the suppression of miR-199a-5p^27^. The same study revealed that miR-199a-5p overexpression significantly increases apoptosis of H9c2 cells and potentiates angiotensin II-induced apoptosis. In this regard, we knew about the relationship between miR199 levels and the progression of HF. Our findings on the effects of miR199a overexpression on cardiac function and hypertrophy are consistent with the results of these studies. We also report a significant increase of miR-199a levels in serum and in the non-infarcted LV versus infarcted LV region (Fig. 1), which is associated with cardiac dysfunction (Table S1) and increased cardiac hypertrophy (Fig. 2). Furthermore, our data indicate that CMs are the main source of miR-199a, as compared to other cardiac cells (Fig. 5). Indeed, data analysis determined that neither CFs nor CECs are able to upregulate miR-199a at least under our experimental conditions. Interestingly, this last result differs from the data published by Joris et al. in 2018^28^. Using bovine aortic endothelial cells, they showed a decrease in the NOS activity after silencing of miR-199a for 48 or 72-h, which they related to an expression of miR-199a in this cellular type^28^. A detailed analysis of these data suggested that it could be related to the experimental conditions used. While they used chronic experimental conditions (i.e. 48 or 72-h), our damage procedure is more acute (i.e. up to 24-h).

In this context, in CMs it is pertinent to highlight differences in miR-199a expression levels over time. Indeed, whereas we evaluated an upregulation in its expression levels in the early stage of damage, this was followed by a significant decrease in the chronic stage (i.e., both 8-weeks and 24 h, in in vivo and in vitro experimental models, respectively). Thus, whether CMs are the main source of miR-199a (Fig. 5), it looks plausible that this decrease is related to a lower cellular viability, which would lead to a release of the cellular content into the extracellular environment (Figs. 1c and 5d). Therefore, extrapolating these results to remote LV area, the CMs are those that upregulate and release miR-199a to the medium at least in the acute stages of damage. Interestingly, these data support the upregulation of miR-199a in non-infarcted LV region, where a population of viable CMs is present. Thus, when we evaluated the modulation of miR-199a in infarcted LV region, where few viable myocytes coincide with other cell types, data analysis showed that miR-199a was not modulating. In addition, we found that endogenous miR199a down-regulation using an antimiR-199a, not only improves cardiac function but also prevents adverse cardiac remodeling (Table S1 and Fig. 2). Based on these results, we next investigated the molecular mechanisms related to the adverse effect of miR-199a on cardiac remodeling following MI. To identify the mechanism of adverse cardiac hypertrophy and miR199a upregulation following MI, we used target prediction algorithms to determine the genes that could be targeted by miR199a. Our analysis revealed that Sirt1 is a potential target of miR199a.

Sirt1 is a nuclear protein that plays a key role in several critical cellular processes including cellular senescence, apoptosis, response to oxidative stress and control of gene expression^29–32^. Sirt1 acts through the deacetylation of a growing number of histones and non-histone proteins^30^. Its expression has been shown to be downregulated in patients with severe HF subsequent to dilated cardiomyopathy, in human left ventricle samples harvested from patients with HF undergoing cardiac transplantation and in peripheral monocytes from patients with either acute coronary syndrome or stable coronary artery disease^33–35^. Emerging evidence from in vitro and in vivo studies indicates that Sirt1 upregulation displays a pro-survival activity in the heart, protects against oxidative damage, inhibits cellular senescence^30–32^ and attenuates cardiomyocyte hypertrophy^36,37^. In the present study, our results are in concordance with the literature confirming that Sirt1 levels, a confirmed direct target of miR-199a^38,39^, are downregulated in the non-infarcted LV region (Fig. 3), which is due to an increase in miR199-5p levels. Our data indicate that increased Sirt1 levels negatively regulate the activity of P300^40,41^. P300 is a transcriptional coactivator that functions as an integrator of numerous signaling pathways, and it regulates many DNA-binding proteins to facilitate transcriptional activation^42^. P300 has been implicated in numerous disease processes, including HF and neurodegenerative diseases^43,44^. In the adult mouse heart, P300 overexpression induces symptoms representative of HF and is accompanied by the acetylation of hypertrophy responsive transcription factors^45^. Genetic reduction of P300 limits both hypertrophy and the attendant risk of HF^46^. Together, these findings imply that in some cases a therapeutic benefit may be obtained by inhibiting P300. The present study demonstrates that P300 expression is increased in failing hearts and CMs under stretching, whereas antimiR199-5p treatment blocks P300 activity by upregulating Sirt1 (Figs. 4 and 7).

Based on these results and given that both Sirt1 and P300 participate in the regulation of cardiac hypertrophy, we hypothesized that these two proteins may act coordinately. Indeed, we identified an interaction between both proteins in basal conditions (Figs. 4 and 7), which favors the deacetylation of P300. As expected, this cardiac effect is reverted following MI where the upregulation of miR199a leads to a decrease in Sirt1 expression level that prevents P300 deacetylation. Interestingly, the use of a specific Sirt1 inhibitor (i.e. EX-527) was able to block the antimiR199a-induced cardioprotective effect on P300 acetylation levels in CMs under stretching (Fig. 7a).

### The miR199/Sirt1/P300 signaling axis regulates cardiac hypertrophy through the upregulation of the circulating soluble sST2 isoform

P300 is also known to take part in multi-molecular complexes in vivo and to function as a co-activator for a variety of genes^47^. Consistently, we demonstrate that P300 co-operates with the transcription factor Yy1 and Sirt1 to regulate sST2 expression following MI. Our experiments, performed in vivo and in vitro, demonstrate the formation of a protein complex between Yy1 and P300 which activates the sST2 transcription promoter (Figs. 4 and 7). Moreover, the deacetylation of P300 by Sirt1 following antimiR199a therapy induces a dissociation of the Yy1-P300 complex, which lead to a decrease of sST2 expression levels after MI. Our results demonstrate the involvement of sST2; indeed, exogenous sST2 addition blocked the cardioprotective effect of anti-miR-199a (Fig. 7l). Therefore, our study indicates that the Sirt1/P300/Yy1/sST2 signaling axis plays an important role in cardiac pathological remodeling after MI.

In summary, our study demonstrates that miR-199a-5p is significantly upregulated after MI, which leads to an increase of circulating sST2 level. Furthermore, miR-199a-5p negatively regulates Sirt1, which leads to P300 acetylation, activation of the transcription factor Yy1 and an increase in the expression and release of circulating soluble ST2 protein. AntimiR199a-5p therapy prevents pathological hypertrophy both in vitro and in vivo after MI. The proposed signal cascade for miR-199a-5p in regulating cardiac pathological hypertrophy following MI is displayed in the supplementary Figure 6. Our study indicates that the miR199a-5p/Sirt1/P300/Yy1/sST2 axis might be a potent therapeutic approach for treating heart failure.

This study has several limitations, which should be pointed out. First, our conclusions are based on data obtained when antimiR-199a therapy is used in acute phase following MI. We do not have information or additional confirmation regarding whether antimiR-199a therapy has similar protective cardiac effect when it is administered in the chronic phase of the disease. The other limitation of our study has been that, despite we have established a relationship between both Yy1 and the genetic modulation of sST2, further detailed analysis is necessary to obtain evidence of any direct interaction between Yy1 transcription factor with the proximal promoter of the *Il1rl1* gen. (CHip assay). Furthermore, it should be considered that the mechanical stretching experimental model used, although it is able to simulate the chronic biomechanical stress that CMs are able to support after MI, does not involve ischemia injury, where other molecular mechanisms could be acting.

## Methods

A detail description of methods is available in the online additional file.

### Animals and ethics statement

12–18-week-old C57Bl/6J male mice (weighing 25–30 g) were purchased from the JACKSON Laboratory. Mice were housed in specific pathogen free environment with a relative humidity of 50 ± 5% at 23 ± 2 °C with 12 h light and dark cycles. Mice had free access to food and water. The study protocol was examined and approved by the Institutional Ethic and Animal Experimentation Committee of the University of Murcia, according to Spanish Government Guidelines and European Community Guidelines for animal care (authorized number A13150105). All methods were carried out in accordance with relevant guidelines and regulations.

### Experimental coronary artery ligation

Animals underwent open chest surgery under general anesthesia using isoflurane (2%) plus O_2_. During surgery, an 8-0 nylon suture was knotted into the anterolateral left ventricular wall around the left anterior descending artery (LAD); the LAD was permanently ligated. After surgery, the animal chest was closed, and the animals were allowed to recover into ventilated racks. Post-operative mortality was 35% during the first 72 h, after which no mice died. Sham-operated mice underwent the same procedure without any ligation.

### LV structure and function

A transthoracic echocardiographic examination was performed in all mice by a blinded trained investigator (MJFP) before surgery (baseline), 24 h post-MI and 4 weeks post-MI. A Vevo 3100 high-frequency ultrasound imaging system (VISUALSONICS, Inc, Toronto, Canada) with a 30-MHz central frequency transducer was used with an integrated rail system III for imaging acquisition. The end-systolic (LVESV), end-diastolic left ventricular (LVEDV) volumes and ejection fraction (EF) were computed using the modified Simpson’s method from the left parasternal long-axis view. Left ventricular (LV) end-diastolic and end-systolic dimensions were also obtained from parasternal short axis M-mode.

### In vivo delivery of LNA-antimiR oligonucleotides and experimental design

Following 24 h of MI, all mice underwent echocardiography to confirm the degree of LV remodeling and cardiac dysfunction. Sham and infarcted mice were then randomized into control or treated groups. The MI groups had a similar degree of LV hypertrophy based on LV wall thickness and LV mass after 24 h of MI, prior to commencement of treatment. Mice were intravenously (caudal vein) administered LNA-control or LNA-antimiR-199a-5p (25 mg/kg/day) over four consecutive days and left for a period of 4 weeks (Fig. 2a). Previous studies demonstrated that four consecutive daily injections of an LNA-antimiR was sufficient to inhibit cardiac miRNA effect for at least 2 months^48^. Animals were randomly divided into six experimental groups: (1) sham mice receiving PBS for 28 days; (2) sham mice receiving antimiR-Ctrl for 28 days; (3) sham mice receiving antimiR-199a for 28 days; (4) infarcted mice receiving PBS for 28 days; (5) infarcted mice receiving antimiR-Ctrl for 28 days; and (6) infarcted mice receiving antimiR-199a for 28 days.

### Tissue samples and histology

Four weeks after the LAD artery ligation, animals were sacrificed, and their hearts were arrested in diastole by intravenous injection of 0.2 ml 10% potassium chloride (MERCK, USA). The heart was excised and rinsed with ice cold DPBS before the removal of the right ventricle and the atria. For the histopathological analyses, mid-papillary slices of the left ventricle of seven mice from each treatment group were fixed in 4% formaldehyde up to 24 h before paraffin embedding. FITC-labeled wheat germ agglutinin (WGA, LIFE TECHNOLOGIES, 1:200 dilution) staining was performed to detect cardiomyocyte cross sectional area in the remote non-infarcted area^48,49^. The cardiac myocyte membranes were observed by fluorescence microscopy. The morphometric analyses were performed using IMAGE-PRO PLUS software. Only cells with well-defined cell membranes were selected. At least one hundred myocytes were analyzed in each group. Masson’s trichrome staining was performed to evaluate fibrosis. For its quantification, at least six random pictures from the remote non-infarcted zone were taken from each slide at 20× magnification. Collagen deposition (blue) was used to define fibrosis, which is expressed as a percentage of blue pixels to red pixels quantitated using *DIGITAL IMAGE HUB software* version 4.0.6. For other molecular-cellular biological studies, both remote non-infarcted as well as infarcted areas were collected and stored at − 80 °C for RNA extraction and Western blot. The observers who performed the images analyses, and the molecular and cellular biological experiments were blinded to the experimental groups.

### Immunohistochemistry

The next experimental procedure was performed as previously described, with some modifications^8^. Briefly, the sections (3 μm) from paraffin-embedded mid-papillary slices of LV were placed on poly-l-lysine-coated glass slides. Then, the sections were de-paraffinized and pre-treated in DAKO PT Link for 20 min at 97 ºC. For Sirt1 or sST2 staining, rehydrated sections were incubated overnight with a polyclonal Sirt1 antibody (working dilution 1:500, PROTEINTECH, Chicago, IL, USA) or with a polyclonal sST2 antibody (working dilution 1:500, PROTEINTECH, Chicago, IL, USA). Next, the sections were incubated with anti-rabbit biotinylated-labelled polymer (DAKO ENVISION) according to the manufacturer's instructions and revealed with 2–2′diaminobencidine (DAB). A cytoplasmic dark-brown precipitate indicated a positive immunostaining. Images were captured using a Zeiss Axio Scope A10 (CARL ZEISS, Madrid, Spain) microscope. Six random pictures were taken of each slide at 10× magnification (n = 7 slices/each treated group).

### Levels of sST2 in serum and cell culture media

The concentrations of sST2 in serum and in the cellular supernatant were measured as previously described^5^; using an ELISA kit (MBS031741; MYBIOSOURCE.COM, San Diego, California, United States). The reaction was terminated using a stop solution, and the absorbance was determined at 450 nm using a microplate reader (CLARIOstar, BMG LABTECK, Ortenberg, Germany). The intra- and inter-assay precisions were < 10%.

### Isolation of primary cells from adult mouse

Wild-type C57BL/6J mice were purchased from JACKSON Laboratory. Hearts of 6–8-week-old mice were harvested and coronary arteries were perfused with buffer A (113 mM NaCl, 4.7 mM KCl, 0.6 mM KH_2_PO_4_, 0.6 mM Na_2_HPO_4_, 1.2 mM MgSO_4_, 12 mM NaHCO_3_, 10 mM KHCO_3_, 10 mM HEPES, and 30 mM taurine) before extracting blood in a retrograde manner by cannulating the aorta. Collagenase type II (WORTHINGTON, Lakewood, N.J.) was added to enzymatically dissociates the ventricular cells. The dissociated cells were then allowed to sediment at 37 °C for 10 min to form a CMs pellet. The CMs were then resuspended in buffer B (47.5 mL of buffer A, 2.5 mL of FCS, and 62.5 µL of 10 mM CaCl_2_). CaCl_2_ was gradually added to the cells to a final concentration of 100 µmol/L, and the isolated CMs were pre-plated in MEM containing 5% FCS, 10 mM 2,3-butanedione monoxime, 2 mM  l-glutamine, and 1% (v/v) penicillin/streptomycin for 2 h at 37 °C and 5% CO_2_. For the CFs, non–myocyte-rich supernatant was then centrifuged at 230×*g* for 5 min, and the pellets were resuspended in MEM containing 5% FCS, 2 mM l-glutamine and 1% (v/v) penicillin/streptomycin and kept initially at 37 °C and 5% CO_2_ for 1 h. Then, culture medium was collected to isolated endothelial cells (see supplementary methods) and attached cells (CFs) were washed three times using pre-warmed PBS and kept at 37 °C and 5% CO_2_ in pre-warmed fresh fibroblasts cell culture medium that has been described previously. To ensure the clean preparation of isolated CMs, CFs, or CECs, the mRNA levels of: (1) β-MHC, c-TnT, and α-actinin (specific CMs markers); (2) vimentin and DDR2 (specific CFs markers); and (3) CD31 (specific endothelial marker) were assessed by quantitative RT-PCR (Supplementary Figure 1). All the assays were conducted 12 h after serum removal to induce cell quiescence. Biomechanical stretching was performed using an adaptation of the experimental design described previously^47–51^. The experimental procedure is described in detail in the additional online methods. When indicated, EX-527 (50 nM) or sST2-Fc (100 nM) were added 30 min before antimiR-199a treatment. The selected concentration of the Sirt1-specific inhibitor EX-527 and its incubation time, does not modulate the levels of expression of miR-199a in CMs (Supplementary Figure 5).

### Luciferase reporter assay

To confirm that miR-199a targets the 3′-UTR of Sirt1, we cloned the region of Sirt1 3′-UTR, which contains the predicted miR-199a binding sites, into a pGL14 Luciferase reporter vector (Luc-Sirt1-WT; PROMEGA, Madison, WI, USA). Luc-Sirt1-mutant (Luc-Sirt1-Mut) was also constructed, which contained a mutated Sirt1 at the binding site of miR-199a (5′-AACACUGG-3′ mutated to 5′-ACCTCTCAA-3′) using In-Fusion cloning kit (CLONTECH, Mountain View, CA, USA) as per manufacturer’s protocol. Dual-Luciferase Reporter Assay System was applied for luciferase reporter assay (PROMEGA). HEK 293T/17 cells (ATCC) was simultaneously transfected with wild type (wt) or mutant (mut) type 3′-UTR Sirt1 and miR-199a mimic or NC mimic. Luciferase activity was detected using CytoFLEX Flow Cytometer (BECKMAN COULTER). Firefly luciferase activity was normalized to Renilla luciferase activity. The ratio of Sirt1 3′-UTR to NC mimic was set as 1; therefore, the displayed results indicate the fold changes relative to control. The experiments were performed three times in triplicate.

### Quantitative real time PCR assay

Total RNA was isolated from both the remote non-infarcted and infarcted myocardial tissue samples as well as CMs under stretching. RNA was purified with the RNeasy Mini Kit (QIAGEN), and cDNA was prepared with the iScript cDNA Synthesis Kit (BIORAD LAB. INC., Madrid) according to the manufacturer’s recommendation. Quantitative real time polymerase chain reaction (RT-qPCR) was performed with the TB Green Premix Ex Taq II (Tli RNase H Plus) Master Mix (TAKARA BIO INC., Europe). Glyceraldehyde 3-phosphate dehydrogenase (GAPDH) was used as housekeeping gene. Sequences of the used primers (MERCK, USA) are listed in additional Table S2.

### Co-immunoprecipitation protocol

For co-immunoprecipitation, each extract from the non-infarcted area (400 μg) or from the cells (8 × 10^6^ cel) were collected and incubated overnight only in the presence of an antibody to acetyl-Lys (ABCAM, ab80178, 1:100), P300 (SANTA CRUZ BIOTECHNOLOGY, sc-48343, 1:100), Sirt1 (CELL SIGNALING TECHNOLOGY, 2310,1:500), or Yy1 (CELL SIGNALING, 2185, 1:500) at 4 °C with gentle shaking. After this, 20 μl of protein A–Sepharose slurry were added and samples were incubated for 4 h at 4 °C with gentle shaking. Then, beads were washed three times with IP buffer (20 mM HEPES pH 7.4, 0.5 mM EDTA, 150 mM NaCl and 0.1% Triton X-100) to remove non-specific binding. For each wash, the beads were mixed gently with IP buffer, centrifuged at 4 °C and the supernatant was discarded. The antigen–antibody complex is eluted from the beads by heating samples in 25 μl 1× SDS gel-loading buffer for 4 min. To analyze the immunoprecipitate by western blot, samples were loaded into a continuous SDS-PAGE gel and run at 30 mA constant current for 2 h. The interaction was analyzed by western blotting using primary antibodies to Sirt1 (CELL SIGNALING TECHNOLOGY, 2310, 1:1000), P300 (SANTA CRUZ BIOTECHNOLOGY, sc-48343, 1:200), Acetyl-Lys (ABCAM, ab80178, 1:100), or Yy1 (CELL SIGNALING, 2185, 1:1000). As control, we used IgG.

### Western blot analysis

Left ventricular samples from the non-infarcted area or from the cells were collected, washed, and lysed with a RIPA solution (THERMOFISHER, USA) supplemented with PMSF (MERCK, USA). The proteins were then extracted, and the proteins concentrations were quantified by BCA^52^. Proteins (35 μg) were denatured, separated by SDS-PAGE electrophoresis and transferred to a polyvinylidene difluoride (PVDF) membrane (MERCK MILLIPORE, USA). The transferred membranes were blocked using 5% bovine serum albumin (BSA) in TBST and incubated with the primary antibodies summarized in the supplementary material. Bands were visualized using enhanced chemiluminescent ECL (AMERSHAM ECL Primer Western Blotting Detection Reagent (GE HEALTHCARE, NJ, USA) (RPN2232)), using a ChemiDoc XRS + system with Image Lab software from BIO-RAD Laboratories (BERKELEY, CA, USA).

### Statistical analyses

Data obtained were reported as mean ± SEM. Statistical differences were evaluated by fitting linear models with interactions (determined by two-way ANOVA followed by Bonferroni’s post hoc test) and estimating marginal means. Holm correction was used for multiple comparisons. A p value < 0.05 was considered significant.

## Perspectives

### Competency in medical knowledge

AntimiR-199a therapy rescues cardiac hypertrophy and heart failure in mice, offering a possible therapeutic approach for cardiac failure. Diseased mice that were treated with antimiR-199a displayed lower levels of sST2 and a better cardiac function.

### Translational outlook

Although an up-regulation of miR199a in the hearts of HF patients has been previously reported, the mechanism of action remains to be understood. Our data suggest that antimiR-199a delivery may have therapeutic value for treating adverse cardiac remodeling following MI. MiR-199a-5p negatively regulates Sirt1, which leads to P300 acetylation, activation of the transcription factor Yy1 and an increase in expression and release of circulating soluble sST2 protein.

## Supplementary Information

Supplementary Information.

## Data Availability

The data that support the findings of this study are available from the corresponding author upon reasonable request.

## Abbreviations

CECs: Cardiac endothelial cells
CFs: Cardiac fibroblasts
CMs: Cardiomyocytes
LV: Left ventricle
MI: Myocardial infarction
P300: E1A-associated nuclear protein P300
Sirt1: Sir-One homolog 1
sST2: Circulating soluble ST2 isoform
ST: Stretching
Yy1: Yin yang 1 transcription factor
