# Supplementary material for: The miRNA199a/SIRT1/P300/Yy1/sST2 signaling axis regulates adverse cardiac remodeling following MI
Source: Sci Rep. 2021 Feb 16;11:3915. doi: 10.1038/s41598-021-82745-9 (PMC7887255; doi:10.1038/s41598-021-82745-9)
Supplement: Supplementary file 1 — Supplementary Information. [file 41598_2021_82745_MOESM1_ESM.doc]

**The miRNA199a/SIRT1/p300/Yy1/sST2 signaling axis regulates adverse cardiac remodeling following MI**

Maria Carmen Asensio-Lopez -PhD^1^, Yassine Sassi-PhD^2^, Fernando Soler-PhD^3^, Maria Josefa Fernandez del Palacio-PhD^4^, Domingo Pascual-Figal-MD, PhD^5^ and Antonio Lax-PhD^1&^.

1. Biomedical Research Institute Virgen de la Arrixaca (IMIB-Arrixaca), University of Murcia, Murcia, Spain.

2. Cardiovascular Research Center, Icahn School of Medicine at Mount Sinai, New York, USA.

3. Biochemical and Molecular Biology Department. University of Murcia, Murcia, Spain.

4. Veterinary Teaching Hospital, Veterinary Medicine and Surgery Department, University of Murcia, Murcia, Spain

5. Cardiology Department, Virgen de la Arrixaca Hospital; IMIB-Arrixaca Biomedical Research Institute; University of Murcia, Murcia, Spain. Spanish National Center for Cardiovascular Research (CNIC); CIBERCV, Madrid, Spain.

&. This author takes responsibility for all aspects of the reliability and freedom from bias of the data presented and their discussed interpretation.

Short running head: AntimiR199-5p prevents myocardial remodeling using the Sirt1/p300/Yy1/sST2 axis as target.

**Address for correspondence:**

1) Antonio Lax Perez, PhD

University of Murcia, Murcia, Spain. Ctra. Madrid-Cartagena s/n, 30120. Tel: +34-868885281; Fax: +34-968369662. E-mail: alax@um.es

2) Domingo A. Pascual-Figal, PhD, MD

Cardiology Department, University of Murcia, LAIB room 2.52, Avda. Buenavista s/n, 30120, Murcia, Spain. Tel: +34-868888136. Fax: +34-968369662. E-mail: dpascual@um.es

**Disclosures:** None

**Funding:** This study was supported by a grant from the Seneca Foundation-Agency of Science and Technology of the Region of Murcia (20652/JLI/18) and a grant from the Instituto de Salud Carlos III (PI19/00519). Dr. Lax is a Ramon and Cajal researcher at the Department of Medicine, University of Murcia.

**ADDITIONAL ONLINE METHODS AND RESULTS**

**Methods.**

*Myocardial infarction model.* All animal experiments were performed in accordance with the regulations adopted by University of Murcia. Adult male C57BL/6J were used as a ligation model of MI (age, 7-8 wks., old; and weight, 22–25 g) and were fed normal rodent chow. Mice were anesthetized with isoflurane (1.5 %) and ventilated through a nose cone with a tidal volume of 0.2 ml at 120 breaths/min using a rodent respirator (model SN-480-7; SHINANO, Tokyo, Japan). The extremity leads of the ECGs were monitored continuously. A thoracotomy was performed in the left third intercostal space, and the beating heart was exposed. An 8-0 polypropylene suture was passed under the left coronary artery at the inferior edge of the left atrium and tied with a slipknot to produce occlusion. Myocardial ischemia was verified by blanching of the LV and ST elevation in ECGs. Air was then evacuated from the chest cavity, and the chest was closed with the ends of the slip outside of the incision. The ventilator was then removed, and normal respiration was restored.

*Bias and Randomization.* All mice were cared for equally in an unbiased fashion by animal technicians and investigators. Mice were randomly allocated into 7 groups (Figure 1) or 6 groups (Figures 2-4). Although the mouse groups were not blinded, the people involved in functional studies on echocardiography, in antimiR-199 dosage, western blot, quantitative PCR and statistical analysis were different, and data were never shared between them up to article writing. In addition, antimiR199 dosage and MI surgery were performed by platform logisticians and both quantitative PCR as well as Western blot by Masters students who were not aware of the nature of the study. Finally, we took care of adding a saline solution condition besides the antagomir scramble control to exclude any bias related to the mouse injection, including the stress associated with mouse handling.

*Echocardiography.* The echocardiographic procedure was performed by a blinded trained investigator (MJFP), following the protocol of the Echocardiography Committee of the Specialty of Cardiology of the American College of Veterinary Internal Medicine and the American Society of Echocardiography recommendations^1,2^. Data are listed in additional file (Table S1).

**Table S1.** Echocardiography measurements of sham and MI control and miR-199a treated mice at 4 weeks post-MI

| Treatment | Sham  +PBS | Sham  +antimiR-Ctrl | Sham  +antimiR-199a | MI  +PBS | MI  +antimiR-Ctrl | MI  +antimiR-199a |
| --- | --- | --- | --- | --- | --- | --- |
| BW (g) | 25.09±0.14 | 25.1±0.33 | 25.67±0.38 | 25.4±0.08 | 25.23±0.2 | 25.51±0.22 |
| HR, bpm | 474.08±9.64 | 451.1±7.07 | 453.3±9.6 | 499.8±10.58 | 443.7±9.09 | 458.67±5.69*** |
| LVEDD (mm) | 4.52±0.05 | 4.51±0.13 | 4.99±0.11 | 4.73±0.04 | 4.76±0.04 | 4.42±0.02* |
| LVESD (mm) | 2.54±0.05 | 2.49±0.07 | 2.75±0.08 | 3.19±0.07 | 3.28±0.05### | 2.62±0.03*** |
| LVEDV (mL) | 70.02±0.25 | 71.75±0.21 | 70.4±1.31 | 103.42±1.54 | 104.25±1.22### | 92.53±2.46*** |
| LVESV (mL) | 33.84±0.64 | 35.14±0.42 | 30.87±0.23 | 67.48±0.59 | 67.65±0.77### | 50.65±1.56*** |
| EF (%) | 51.68±0.87 | 51.02±0.57 | 56.01±0.86 | 34.58±1.34 | 34.99±1.29### | 45.28±0.71*** |
| FS (%) | 43.69±1.14 | 44.86±0.73 | 44.99±0.92 | 32.61±1.61 | 31.08±1.14### | 40.62±0.83*** |

BW, body weight; HR, heart rate; LV, left ventricle; LVEDV, left ventricular end-diastolic volume; LVESV, left ventricular end-systolic volume; LVEDD, left ventricular dimensions at end diastole; LVESD, left ventricular dimensions at end systole; FS, fractional shortening; EF, ejection fraction. ###P<0.001 versus Sham+antimiR-Ctrl, *P<0.05 and *** p<0.001 versus MI+antimiR-Ctrl. P values were calculated using unpaired, two-tailed Student’s t-tests. Data represent means ± SEM.

*LNA oligonucleotides.* The miRCURY LNA microRNA Inhibitor (mmu-miR-199a-5p and a scrambled control) is LNA-enhanced (LNA/DNA mixmer with ~50% LNA content) and contains a phosphorothioate backbone (EXIQON, Skelstedet, Denmark). LNA-oligonucleotides were purified and analyzed using anion-exchange high-performance liquid chromatography, desalted and lyophilized as a sodium salt. LNA-oligonucleotides were resuspended in saline at 5 mg/ml, aliquoted and stored at −20 °C. The sequence for mmu-miR-199a-5p antimiR is 5′ CCCAGUGUUCAGACUACCU3′ (Batch Number YI04101097) and for the scrambled control 5′ACGTCTATACGCCCA 3′ (Batch Number 508005).

*Isolation of cardiac endothelial cells.* Non–myocyte-rich supernatant obtained after isolation of CMs (see methods section in main manuscript) was centrifuged at 250 xg for 10 min. The pellet was resuspended in HBSS supplemented with 2 mM EDTA and 0.25 % BSA, before their purification by flow cytometry (Sorting). For FACS, we used the specific endothelial cells antibody anti-CD31 of BD BIOSCIENCES to their purification (Catalog Nº: 558738). Briefly, the antibody (5 µl) was incubated with the cells (1x10^6^) for 30 min at 4 ºC in HBSS. We sorted live and single cell-gated subpopulations based on their staining patterns with FITC-anti-CD31 using a FACS Aria II Cell Sorter and FACS Diva vs. 6.1(BD BIOSCIENCES). FACS analyses were conducted using the FlowJo software package (TREE STAR, Ashland, OR). The sorted cells were collected in MV2 endothelial cell culture medium according to manufacturer´s instructions. Isolated cells were allowed to attach overnight in a CO_2_ incubator and the culture medium was changed every 2 days.

*Biomechanical strain model.* To induce biomechanical strain the experimental protocols described by Banerjee et al.^3^, with some modifications were followed. Briefly, primary cardiomyocytes, cardiac fibroblasts or endothelial cells were cultured in six-well Flex I culture plates (BF-3001C FLEXCELL INTERNATIONAL, USA) coated with collagen I. All the assays were conducted in absence of serum. The biomechanical stretch was conducted using a Flexcell FX-5000 Tension system (FLEXCELL INTERNATIONAL, McKeesport, PA). This device uses a controlled vacuum to deform the monolayer of cells grown on top of the membrane. In or case, the vacuum produced a 16% elongation on the flexible bottom elastomer membranes at a frequency of 60 cycles/min (1 Hz). Control cells were also plated on Bioflex plates to avoid variations based on attachment stratum and in static conditions.

*RNA isolation.* Total RNA was isolated from frozen mouse tissues using TRI Reagent (MERCK, USA) and quantitated on a Nanodrop Spectrometer Thermo Scientific, WALTHAM, MA, USA).

*Quantitative RT-PCR (qPCR).* For mRNA expression analysis, 2 μg of total RNA was reverse transcribed using the High Capacity RNA-to-cDNA kit (LIFE TECHNOLOGIES, Carlsbad, CA, USA) according to manufacturer’s recommendations. qPCR was performed using TaqMan probes (LIFE TECHNOLOGIES) and amplified on an Applied Biosystems 7500 real-time PCR instrument according to manufacturer’s instructions. Glyceraldehyde-3-Phosphate Dehydrogenase (GAPDH) was used to standardize for cDNA concentration and data was analyzed using the 2−∆∆Ct method of quantification. For miRNA expression analysis, 50–100 ng of total RNA was reversed transcribed using TaqMan MicroRNA Reverse Transcription Kit (LIFE TECHNOLOGIES) according to manufacturer’s recommendations. qPCR was performed using TaqMan MicroRNA Assays (LIFE TECHNOLOGIES), expression was normalized against snoU6 and data analyzed using the 2−∆∆Ct algorithm. The amplified genes and primer sequences are described in online table S2

BNP, brain natriuretic peptide; Col, collagen; GAPDH, glyceraldehyde-3-phosphate; dehydrogenase; GCH1, cardiac GTP cyclohydrolase 1; Myh, myosin heavy chain; Nppa, natriuretic peptide precursor A. PTX, pentraxin 3; JunB, jun B proto-oncogene.

*SDS-Polyacrylamide gel electrophoresis (PAGE) and Western blotting.* Heart protein lysate (20–100 μg) was separated by SDS-PAGE, transferred to PVDF membrane and probed overnight with primary antibodies for GAPDH, (MERCK, 8795,1:5000), Sirt1 (CELL SIGNALING TECHNOLOGY, 2310,1:1000), P300 (SANTA CRUZ BIOTECHNOLOGY, sc-48343, 1:200), Acetyl-Lys (ABCAM, ab80178, 1:100), Yy1 (CELL SIGNALING, 2185,1:1000), and TBP (CELL SIGNALING, 8515, 1:1000). Signals were detected by chemiluminescence and quantified using ImageJ 1.44p pixel analysis (US National Institutes of Health). Nuclear extracts were isolated using procedures described previously^7^.

**Results**


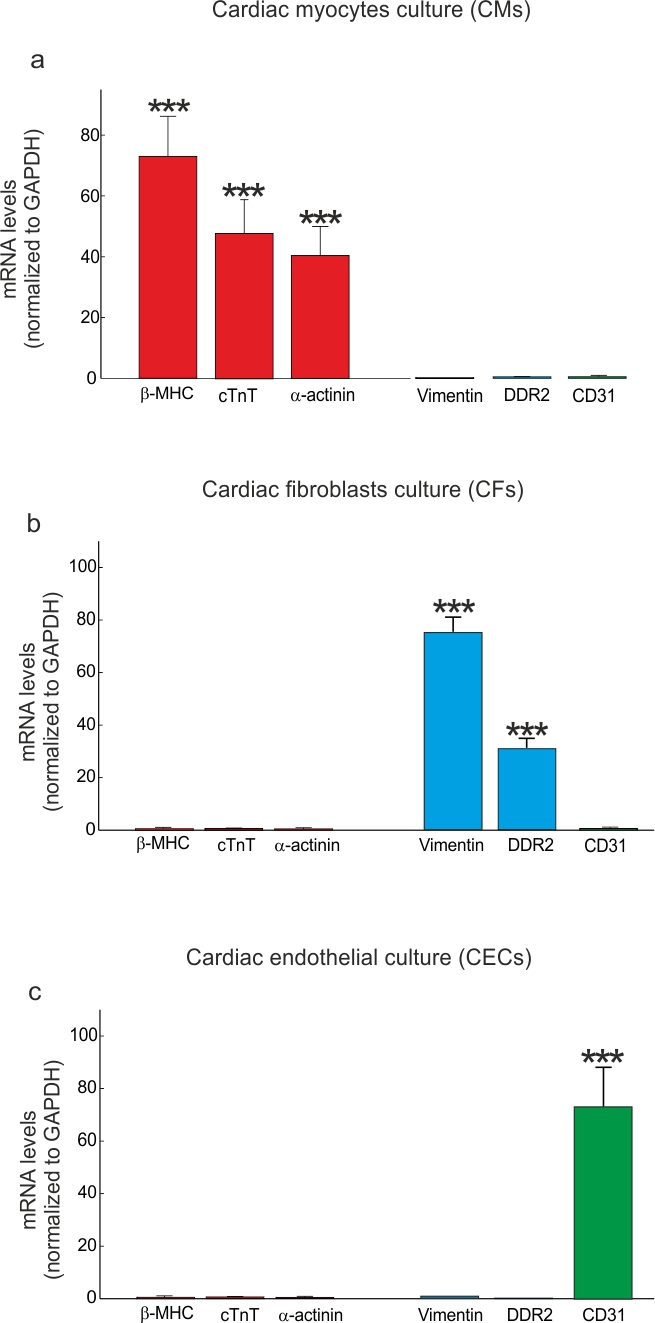
1.- *Purity of primary cells fractions.* Analysis of β-MHC, c-TnT and α-actinin mRNA levels in cardiac myocytes cultures by RT-qPCR, were significantly upregulated with regard to their respective expression levels in the other primary cultures (p<0.001 in all cases) (Supplementary figure 1a). When we evaluated the purity of isolated primary cardiac fibroblasts, mRNA levels of vimentin and DDR2 were significantly higher (p<0.001, for all markers) as compared to both primary CMs and CECs cultures (Supplementary figure 1b). Finally, CD31 mRNA expression levels was significantly higher in cardiac endothelial cell culture as compared to both primary CMs and CFs cultures (p<0.001) (Supplementary figure 1c).

**Supplementary figure 1.**

**Purity of primary cardiomyocytes, fibroblasts and cardiac endothelial culture.** mRNA levels for: (1) β-MHC, c-TnT, and α-actinin as cardiomyocytes-specific markers; (2) vimentin, and DDR2 as cardiac fibroblast-specific markers; and (3) CD31 as endothelial-specific marker, were analyzed by quantitative RT-PCR. All quantifications derive from n = 10 independent assays/group. All quantitative data are reported as means ± SEM. ***p < 0.001 determined by one-way ANOVA and multiple comparisons according to cell culture were performed to verify that the expression was produced only in its specific primary culture and not in the others. CMs: cardiomyocytes; CFs: cardiac fibroblasts; CECs: cardiac endothelial cells; c-TnT: cardiac troponin T; β-MHC: beta-cardiac myosin heavy chain; DDR2: discoidin domain receptor 2.

2.- Upon isolation of CMs and CFs from non-infarcted LV region of infarcted mice, miR-199a were significantly higher in CMs compared to CFs at 4 weeks after MI (Supplementary figure 2).

**
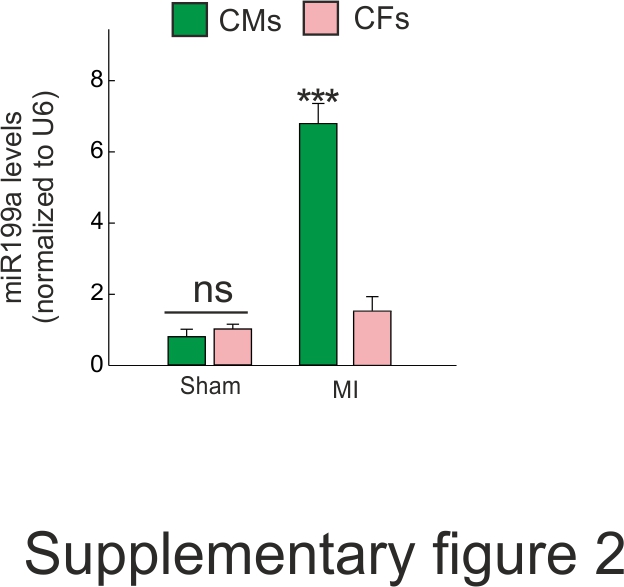
**

**Supplementary Figure 2.**

**miR-199a levels were higher in CMs compared to CFs.** Determination of miR199a by RT-qPCR from primary cardiac myocytes and fibroblasts isolated from the hearts of infarcted mice 28 days after MI; n=5 mice per groups. All quantitative data are reported as means ± SEM. ***p<0.001 CMs vs. CFs. CFs: cardiac fibroblasts; CMs: cardiomyocytes; MI: myocardial infarction; ns: non-significant.


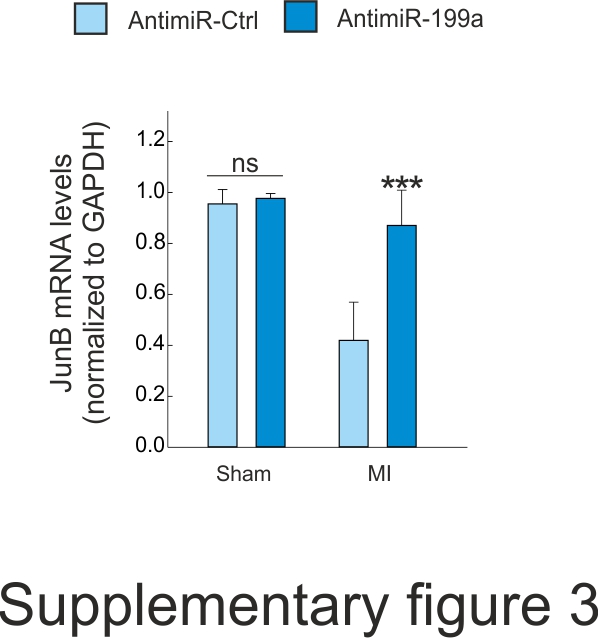
*3.- AntimiR-199a therapy increases JunB mRNA levels in CMs isolated from the LV remote region.* AntimiR-199a therapy leads to lead to increase of 56% in JunB mRNA levels after 4 weeks following MI (p<0.001) in compared to groups treated with antimiR-Ctrl.

**Supplementary figure 3.**

**AntimiR199a therapy increases JunB mRNA levels in remote non-infarcted myocardium.** Quantitative real-time PCR analysis of JunB in non-infarcted myocardium; n=5 mice per groups. All quantitative data are reported as means ± SEM. *** p<0.001 respect to Sham+antimiR-Ctrl therapy. Abbreviations as in supplementary figure 2.


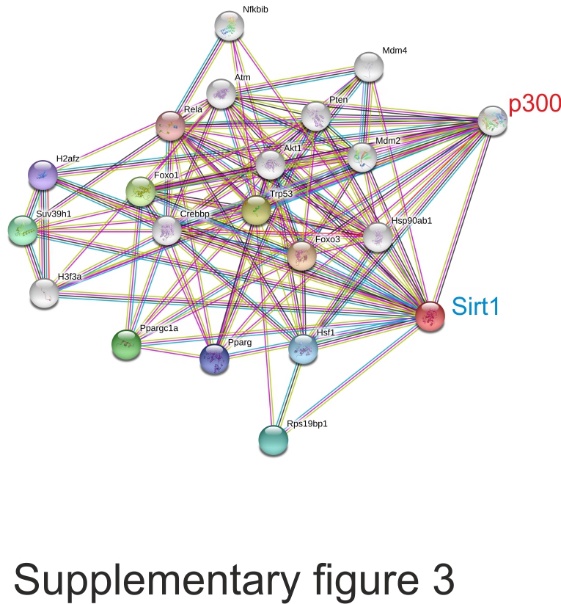
*4.- Sirt1 protein and p300.* Proteomic analysis of the Sirt1 interactome, we identified and confirmed that Sirt1 interacts with p300 in murine hearts with a score of 0.93 (Supplementary figure 4).

**Supplementary Figure 4.**

**Sirt1 interacts with p300 in murine hearts.** Data analysis obtained from STRING database (http://string-db.org/), allowed us to determine that Sirt1 interacts with P300 in the murine heart with a score of 0.93.


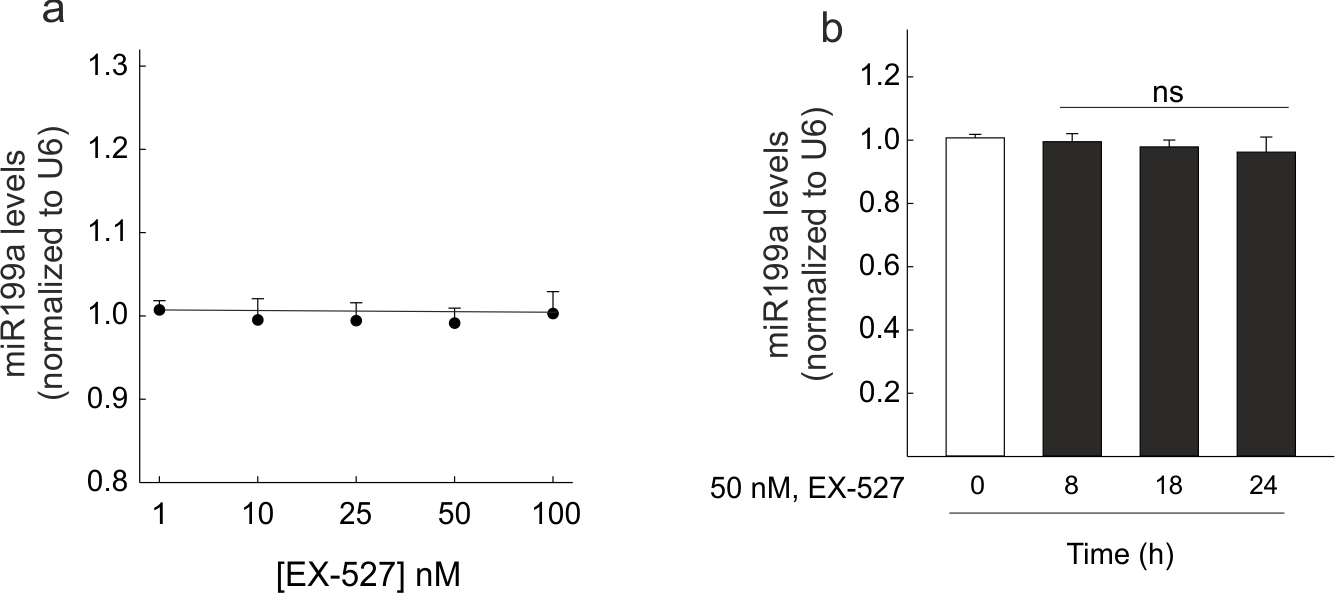
*5. Under our experimental conditions, the Sirt1-specific inhibitor EX-527 did not affect at miR-199a expression levels.* As shown, no significant differences were observed when cardiomyocytes were treated with a given EX-527 concentration for 18 h (supplementary figure 5a). Furthermore, no difference over time was evaluated for the miR-199a levels, when cardiomyocytes were treated with 50 nM EX-527 (supplementary figure 5b).

**Supplementary Figure 5.**

**Effect of EX-527 on miR-199a expression levels.** (a) Mir-199a expression levels in cardiomyocytes treated with a given EX-527 concentration for 18h. (b) MiR-199a expression levels in cardiomyocytes treated with 50 nM EX-527 after different incubation times. All quantifications derive from n = 10 independent assays/group. All quantitative data are reported as means ± SEM. ns: non-significant.

6. Representative proposed signal cascade for miR-199a-5p in regulating cardiac pathological hypertrophy following MI


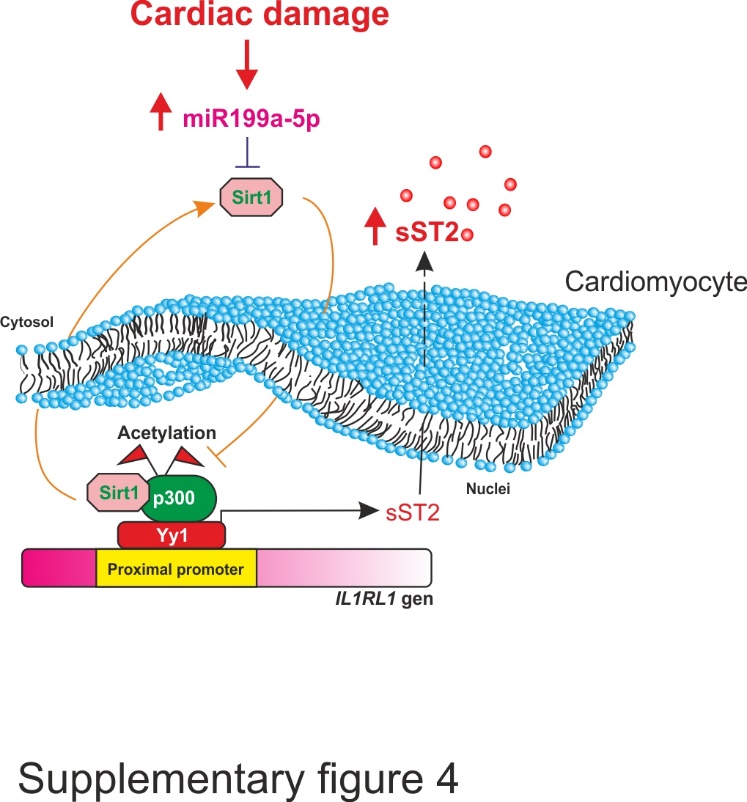


**Supplementary Figure 6**

**Representative scheme miR199a/Sirt1/p300/Yy1/sST2.** miR-199a-5p is significantly upregulated after MI and increases pathological hypertrophy by increasing circulating sST2 levels. Furthermore, miR-199a-5p negatively regulated Sirt1, which lead to P300 acetylation, activation of the transcription factor Yy1 and an increase in the expression and release of circulating soluble sST2 protein. Although our study has no studied proximal promoter activity others published studies have described its specific relation to sST2 expression^4-8^.

**Representative full Western Blots**

**
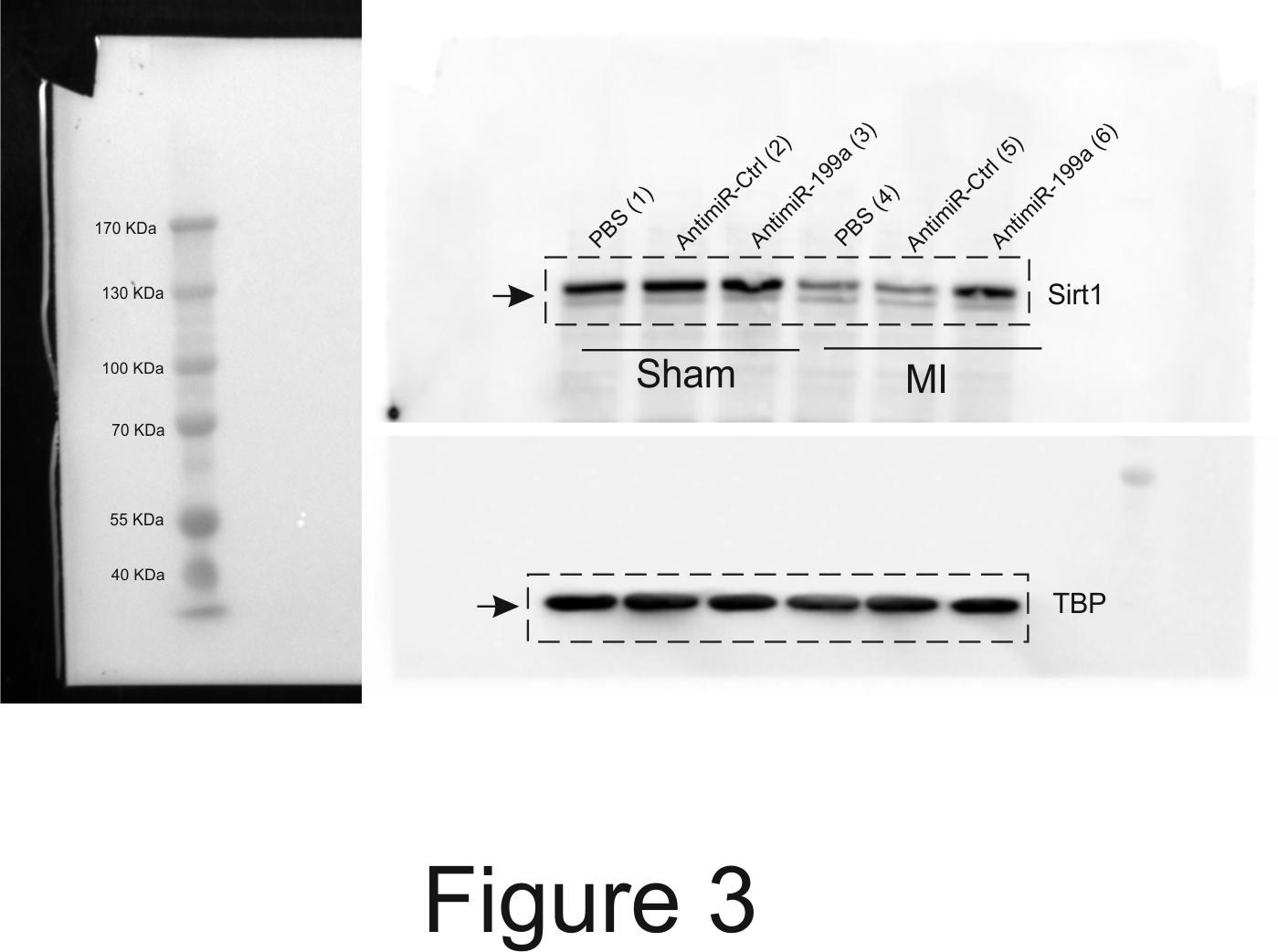
**

**Supplementary Figure 7.**

*Representative Full Western Blot images for Figure 3.* Blots cropped from different parts of the same gel. The cropped blots are displayed using a discontinuous blue line.

**
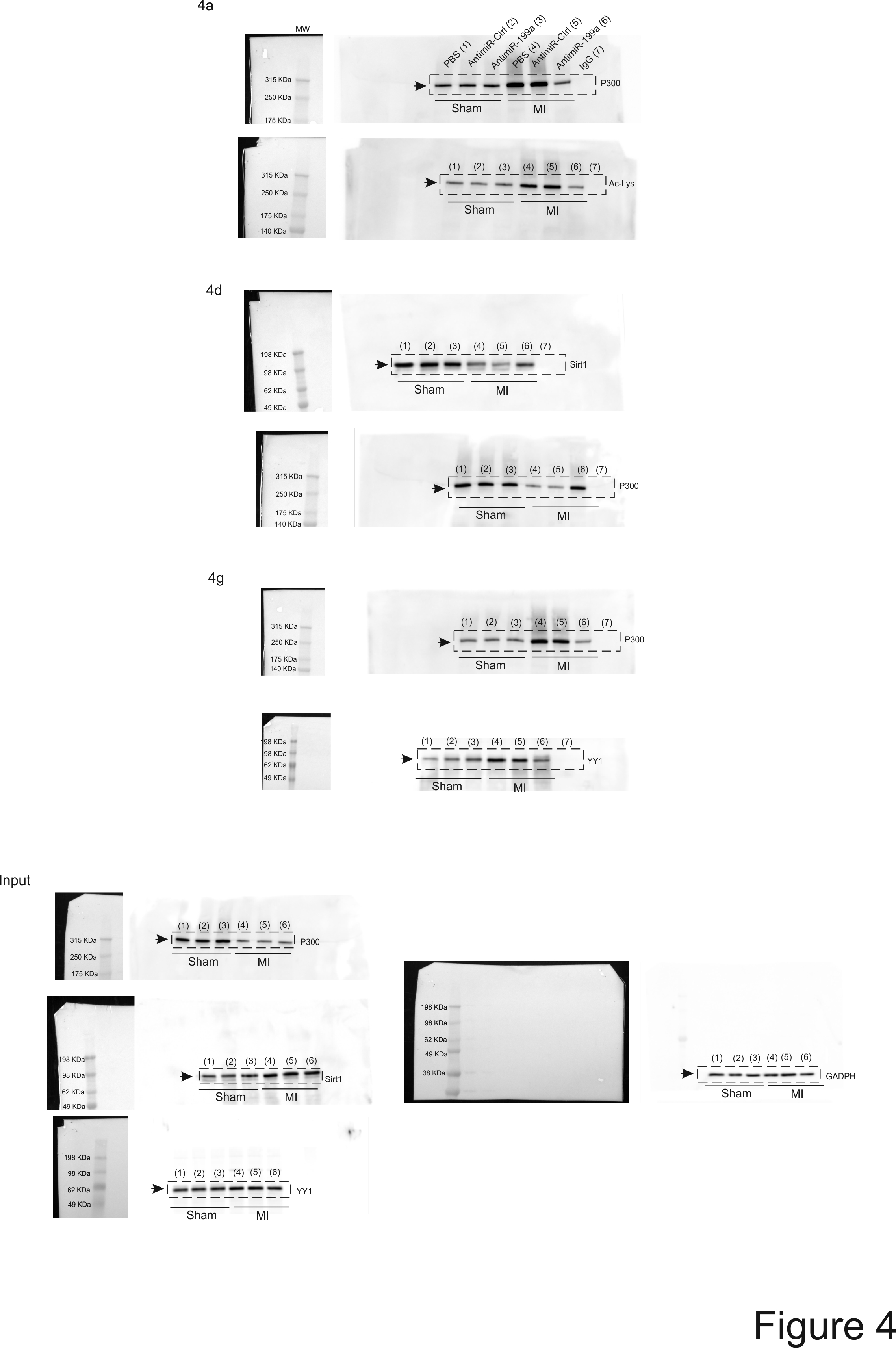
**

**Supplementary Figure 8.**

*Representative Full Western Blot images for Figure 4.* The cropped blots are displayed using a discontinuous blue line. Independent blots images; cropped each them from different gel.

**
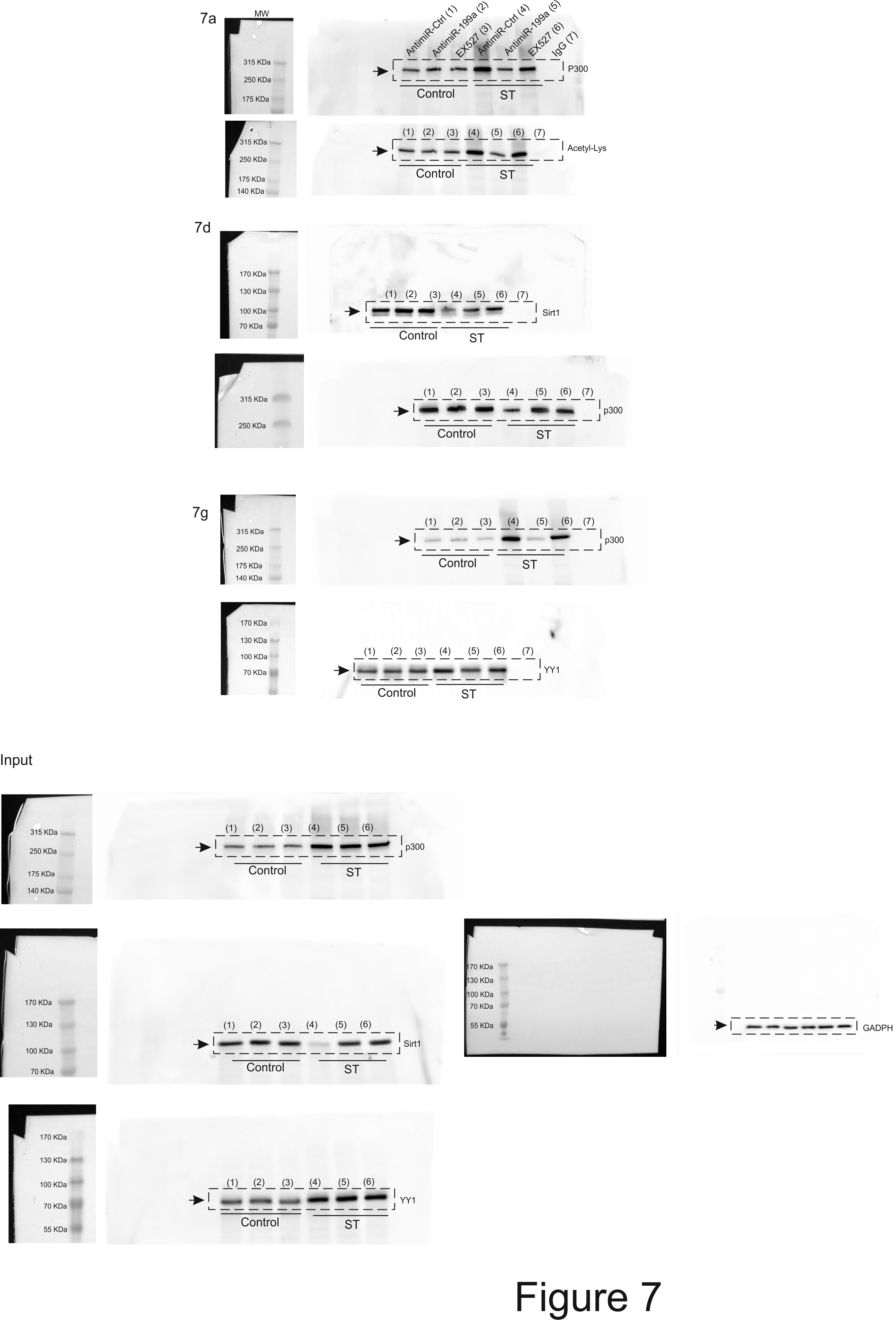
Supplementary Figure 9.**

*Representative Full Western Blot images for Figure 7.* The cropped blots are displayed using a discontinuous blue line. Independent blots images; cropped each them from different gel.

**Data from figure 1**

|  | **Treatment** | | | | | | |
| --- | --- | --- | --- | --- | --- | --- | --- |
|  | sham | MI_1d | MI_2d | MI_1w | MI_2w | MI_4w | MI_8w |
| n | 7 | 7 | 7 | 7 | 7 | 7 | 7 |
| miR199a_Non-Infarcted LV  myocardium | 1.05±  0.17 | 0.63±  0.08 | 4±  0.36 | 9.01±  1.02 | ---- | 8.87±  1.71 | 4.49±  1.42 |
| miR199a_Infarcted LV  myocardium | 1.04±  0.05 | ---- | ---- | 1.19±  0.28 | ---- | 1.14±  0.34 | ---- |
| miR199a_Serum | 1.02±  0.11 | ---- | ---- | 0.8±  0.08 | 1.83±  0.39 | 5.24±  0.52 | 15.83±  3.02 |

Data from figure 2

|  | Treatment | | | | | |
| --- | --- | --- | --- | --- | --- | --- |
|  | Sham+  PBS | Sham+  AC | Sham+  AM199a | IM+  PBS | IM+  AC | IM+  AM199a |
| n | 12 | 10 | 10 | 10 | 10 | 9 |
| JunB mRNA levels | 0.9±0.1 | 1.02±0.1 | 1±0.19 | 0.39±0.05 | 0.46±0.04 | 0.87±0.06 |
| FS (%) | 43.69±  1.14 | 44.86±0.73 | 44.99±0.92 | 32.61±1.61 | 31.08±1.14 | 40.62±0.83 |
| HW/TL | 6.97±  0.38 | 7.06±0.33 | 7.48±0.23 | 9.63±0.57 | 9.65±0.36 | 7.56±0.41 |
| Fibrosis (%) | 4.88±  0.23 | 5.13±0.28 | 5.26±0.27 | 14.26±0.41 | 13.07±0.87 | 7.89±0.42 |
| CM area | 216.92±4 | 242±5.34 | 249.1±4.14 | 400±10.35 | 404.4±10.57 | 287.44±7.79 |
| Myh7/Myh6 mRNA levels | 1±  0.02 | 1±0.03 | 1±0.02 | 7.71±0.09 | 7.79±0.09 | 3.75±0.13 |
| Nppa mRNA levels | 1.02±  0.03 | 1±0.02 | 0.99±0.01 | 4.56±0.23 | 4.65±0.35 | 2.22±0.12 |
| Col1a1 mRNA levels | 1.01±  0.02 | 1±0.02 | 0.97±0.02 | 55.59±0.61 | 56.58±0.52 | 21.25±0.59 |
| Col3a1 mRNA levels | 1±0.03 | 1±0.02 | 1±0.02 | 66.41±0.65 | 64.88±1.34 | 43.46±1.42 |

Data from figure 3b

|  | **Treatment** | | | |
| --- | --- | --- | --- | --- |
|  | Control+AC | Control+mimic | Mut+AC | Mut+mimic |
| n | 10 | 10 | 11 | 11 |
| luciferase_activity | 6.37±0.03 | 3.13±0.18 | 6.52±0.11 | 6.43±0.06 |

**Data from figure 3, c-e**

|  | **Treatment** | | | | | |
| --- | --- | --- | --- | --- | --- | --- |
|  | Sham+  PBS | Sham+  AC | Sham+  AM199a | IM+  PBS | IM+  AC | IM+  AM199a |
| n | 12 | 10 | 10 | 10 | 10 | 9 |
| Sirt1 mRNA levels | 1±0.01 | 1±0.02 | 1±0.02 | 0.43±0.02 | 0.43±0.03 | 0.77±0.02 |
| Sirt1 protein levels | 3.87±0.08 | 3.85±0.08 | 3.81±0.05 | 1.53±0.03 | 1.4±0.04 | 2.85±0.05 |
| Sirt1 levels_IHC | ---- | 7.53±0.2 | 7.39±0.14 | ---- | 2.64±0.08 | 5.44±0.24 |

**Data from figure 4**

|  | **Treatment** | | | | | |
| --- | --- | --- | --- | --- | --- | --- |
|  | Sham+  PBS | Sham+  AC | Sham+  AM199a | IM+  PBS | IM+  AC | IM+  AM199a |
| n | 12 | 10 | 10 | 10 | 10 | 9 |
| Panel b | 1±0.01 | 0.99±0.02 | 1±0.01 | 2.57±0.06 | 2.69±0.07 | 1.32±0.06 |
| Panel c | 1.01±0.01 | 1±0.01 | 1±0.01 | 3.6±0.1 | 3.5±0.09 | 1.32±0.05 |
| Panel e | 1±0.01 | 1±0.01 | 1±0.01 | 0.45±0.02 | 0.43±0.02 | 0.83±0.01 |
| Panel f | 1±0.01 | 1±0.01 | 1±0.01 | 0.62±0.02 | 0.61±0.02 | 0.89±0.02 |
| Panel h | 1±0.01 | 1±0.01 | 1±0.01 | 2.51±0.04 | 2.58±0.01 | 1.25±0.04 |
| Panel i | 0.99±0.01 | 1±0.01 | 0.99±0.01 | 3.96±0.08 | 3.82±0.1 | 1.54±0.04 |
| sST2 levels_IHC | ---- | 0.97±0.02 | 0.99±0.01 | ---- | 5.78±0.09 | 2.55±0.03 |

**Data from figure 5**

|  | **Experiments** | | | | |
| --- | --- | --- | --- | --- | --- |
|  | Control | ST 8 h | ST 12 h | ST 18 h | ST 24 h |
| n | 12 | 10 | 10 | 10 | 10 |
| miR199a levels_CMs | 1±0.01 | 1.21±0.02 | 2.53±0.07 | 3.86±0.08 | 1.7±0.07 |
| miR199a levels_CFs | 1±0.01 | 0.98±0.01 | 1.09±0.01 | 1.01±0.01 | 1.01±0 |
| miR199a levels_Culture medium from CMs | 1±0.01 | 0.99±0.01 | 3.9±0.06 | 6.33±0.1 | 12.1±0.14 |
| miR199a levels_Culture medium from CFs | 1.01±0.01 | 1.01±0.01 | 0.97±0.01 | 1.02±0.01 | 1±0.01 |

**Data from figure 6**

|  | **Experiments** | | | | | |
| --- | --- | --- | --- | --- | --- | --- |
|  | Control+  PBS | Control+  AC | Control+  AM199 | ST+  PBS | ST+  AC | ST+  AM199 |
| n | 10 | 10 | 10 | 10 | 10 | 10 |
| JunB mRNA levels | 1±0 | 1±0.01 | 1±0.01 | 0.48±0.02 | 0.46±0.02 | 0.88±0.02 |
| Sirt1 mRNA levels | 1.01±0 | 1±0.01 | 1±0.01 | 0.48±0.02 | 0.46±0.01 | 0.72±0.02 |
| Cellular viability (%) | 99.5±0.43 | 99.3±0.33 | 100.1±0.43 | 43.4±0.45 | 38.3±0.84 | 74.2±0.76 |
| LDH levels | 0.99±0.01 | 1±0.01 | 1±0.01 | 3.54±0.02 | 3.54±0.04 | 1.51±0.03 |
| Myh7/Myh6 mRNA levels | 1±0.01 | 0.99±0.01 | 1±0.01 | 8.08±0.04 | 8.79±0.09 | 4.28±0.03 |
| Nppa mRNA levels | 1±0.01 | 1±0 | 0.99±0.01 | 4.51±0.02 | 4.58±0.02 | 2.31±0.04 |
| BNP mRNA levels | 1±0.01 | 0.99±0.01 | 1±0.01 | 4.25±0.04 | 4.42±0.02 | 2.48±0.04 |

**Data from figure 7a-i**

|  | **Experiments** | | | | | |
| --- | --- | --- | --- | --- | --- | --- |
|  | Control+  AC | Control+  AM199 | Control+AM199+ EX527 | ST+  AC | ST+  AM199 | ST+AM199  +EX527 |
| n | 10 | 10 | 10 | 10 | 10 | 10 |
| Panel b | 1±0.01 | 1±0.01 | 0.99±0.01 | 2.97±0.03 | 1.25±0.04 | 2.27±0.01 |
| Panel c | 1±0.01 | 0.96±0.02 | 1±0.01 | 4.34±0.04 | 1.51±0.01 | 3.27±0.02 |
| Panel e | 1±0.01 | 1.01±0.01 | 0.99±0 | 0.53±0.01 | 0.8±0.01 | 0.81±0.01 |
| Panel f | 0.99±0.02 | 1±0.01 | 1±0.01 | 0.45±0.01 | 0.91±0.02 | 0.82±0.01 |
| Panel h | 1.01±0.02 | 1±0 | 1±0.01 | 2.56±0.02 | 1.6±0.02 | 2.38±0.03 |
| Panel i | 1±0.01 | 0.99±0.01 | 1±0.01 | 3.62±0.07 | 2.3±0.04 | 3.17±0.03 |

**Dato from figure 7j-l**

|  | **Experiments** | | | | |
| --- | --- | --- | --- | --- | --- |
|  | Control+  AC | ST+  AC | ST+  AM199 | ST+AM199  +EX527 | ST+AM199  +sST2-Fc |
| n | 10 | 10 | 10 | 10 | 10 |
| sST2 levels in culture medium | 16.05±1.09 | 43.63±1.37 | 19.89±1.33 | 41.45±0.72 | ---- |
| Myh7/Myh6 mRNA levels | ---- | 8.11±0.06 | 4.71±0.07 | ---- | 8.23±0.13 |
| Nppa mRNA levels | ---- | 4.51±0.04 | 2.29±0.04 | ---- | 4.07±0.06 |
| PTX3 mRNA levels | ---- | 3.88±0.05 | 1.95±0.02 | ---- | 4.11±0.03 |
| LDA levels | ---- | 4.01±0.02 | 1.87±0.02 | ---- | 3.72±0.06 |

**References**

1. Sahn, D. J., DeMaria, A., Kisslo, J. & Weyman, A. Recommendations regarding quantitation in M-mode echocardiography: results of a survey of echocardiographic measurements. *Circulation* **58**, 1072–83 (1978).

2. Thomas, W. P. *et al.* Recommendations for standards in transthoracic two-dimensional echocardiography in the dog and cat. Echocardiography Committee of the Specialty of Cardiology, American College of Veterinary Internal Medicine. *J. Vet. Intern. Med.* **7**, 247–52

3. Banerjee, I. *et al.* Cyclic stretch of embryonic cardiomyocytes increases proliferation, growth, and expression while repressing Tgf-β signaling. *J. Mol. Cell. Cardiol.* **79**, 133–144 (2015).

4. Shimizu, M. *et al*., Functional SNPs in the distal promoter of the ST2 gene are associated with atopic dermatitis. *Hum Mol Genet.* **14**, 2919-27 (2005).

5. Griesenauer B, Paczesny S. The ST2/IL-33 Axis in Immune Cells during Inflammatory Diseases. Front Immunol. 2017; 8:475. (2017).

6. Bonyadi rad, e., et al., Runx2 mediated Induction of Novel Targets ST2 and Runx3 Leads to Cooperative Regulation of Hypertrophic Differentiation in ATDC5 Chondrocytes. **7**. Sci Rep. 17947. (2017)

7. Asensio-Lopez, MC et al. Yin-Yang 1 transcription factor modulates ST2 expression during adverse cardiac remodeling post-myocardial infarction. J Mol Cell Cardiol. **130**, 216-233, (2019).

8. Weinberg EO, et al. Expression and Regulation of ST2, an Interleukin-1 Receptor Family Member, in Cardiomyocytes and Myocardial Infarction. Circulation. **106**, 2961–66, (2002).
